# Optimizing Spatio-Temporal Allocation of the COVID-19 Vaccine Under Different Epidemiological Landscapes

**DOI:** 10.3389/fpubh.2022.921855

**Published:** 2022-06-23

**Authors:** Wen Cao, Jingwen Zhu, Xinyi Wang, Xiaochong Tong, Yuzhen Tian, Haoran Dai, Zhigang Ma

**Affiliations:** ^1^Department of Remote Sensing and Geographic Information Science, School of Geoscience and Technology, Zhengzhou University, Zhengzhou, China; ^2^Department of Photogrammetry and Remote Sensing, School of Geospatial Information, University of Information Engineering, Zhengzhou, China; ^3^PIESAT Institute of Applied Beidou Navigation Technologies at Zhengzhou, Zhengzhou, China

**Keywords:** COVID-19, epidemiological landscapes, optimal vaccine allocation, policy decision, vaccination

## Abstract

An efficient and safe vaccine is expected to allow people to return to normal life as soon as possible. However, vaccines for new diseases are likely to be in short supply during the initial deployment due to narrow production capacity and logistics. There is an urgent need to optimize the allocation of limited vaccines to improve the population effectiveness of vaccination. Existing studies mostly address a single epidemiological landscape. The robustness of the effectiveness of other proposed strategies is difficult to guarantee under other landscapes. In this study, a novel vaccination allocation model based on spatio-temporal heterogeneity of epidemiological landscapes is proposed. This model was combined with optimization algorithms to determine the near-optimal spatio-temporal allocation for vaccines with different effectiveness and coverage. We fully simulated the epidemiological landscapes during vaccination, and then minimized objective functions independently under various epidemiological landscapes and degrees of viral transmission. We find that if all subregions are in the middle or late stages of the pandemic, the difference between the effectiveness of the near-optimal and pro-rata strategies is very small in most cases. In contrast, under other epidemiological landscapes, when minimizing deaths, the optimizer tends to allocate the remaining doses to sub-regions with relatively higher risk and expected coverage after covering the elderly. While to minimize symptomatic infections, allocating vaccines first to the higher-risk sub-regions is near-optimal. This means that the pro-rata allocation is a good option when the subregions are all in the middle to late stages of the pandemic. Moreover, we suggest that if all subregions are in the period of rapid virus transmission, vaccines should be administered to older adults in all subregions simultaneously, while when the epidemiological dynamics of the subregions are significantly different, priority can be given to older adults in subregions that are still in the early stages of the pandemic. After covering the elderly in the region, high-risk sub-regions can be prioritized.

## Introduction

The rapid spread of the severe acute respiratory syndrome coronavirus 2 (SARS-CoV-2) has triggered a public health and economic crisis worldwide. As of January 15, 2022, there have been more than 3.1 billion cases and 5.5 million deaths reported (1). To combat this crisis, authorities have implemented various non-pharmaceutical interventions according to local conditions. Both empirical analysis and mathematical modeling suggest that non-pharmacological interventions, particularly lockdown and strict quarantine measures (2–4), are essential to mitigate the spread of the virus in the short term. However, given the high socio-economic costs of such measures, a long-term solution—an effective and safe vaccine-remains urgently needed. With the successive implementation of mass vaccination campaigns, the huge demand for vaccines has left many countries and regions facing severe resource shortages and supply imbalances (5, 6). Optimizing the allocation of limited vaccines is an urgent and critical issue for all countries.

The mathematical models help to inform the public and policymakers about possible scenarios for the development of infectious diseases and the potential effectiveness of different intervention methods. The compartmental models can be easily used to simulate various interventions. Epidemiological parameters in these models and simulation results are easy to understand. Based on the characteristics of this COVID-19 pandemic, a series of extensions for the classical SIR model have been proposed, such as age-group separation (7, 8), the introduction of asymptomatic infections (9, 10), deaths (11), and immunity period (12), and the incorporation of graph-based spatial components (10, 13). The efficacy and effectiveness of most vaccines on various endpoints have been assessed through rigorous controlled clinical trials or observational studies, such as BNT162b2 (14, 15), ChAdOx1 (16), mRNA-1273 (17), and inactivated SARS-CoV-2 vaccine (18). The optimal allocation of vaccines involves three main dimensions: object, timing, and places. Most of such studies focused on to whom and suggested that vaccine should be prioritized to people by age and risk. For the individuals in a region, prioritized vaccination of the elderly is beneficial in reducing severe symptomatic infections and deaths, while vaccinating younger age groups first can minimize symptomatic infections (11, 12, 19–21). The research on the spatio-temporal allocation of vaccines can be divided into two parts. Some studies used mathematical models to evaluate the effectiveness of different allocation strategies (22–26). Another approach was using optimization algorithms to find the possible optimal strategy (27–29). In existing studies, only the allocation for the epidemiological landscape (mainly refers to epidemic dynamics in sub-regions in our work) of a region over a certain period was identified. This does help to address the vaccine allocation under the corresponding epidemiological landscape. However, epidemiological landscapes change over time, and different countries may face different epidemiological landscapes when deploying vaccines. Once the epidemiological landscape changes, the effectiveness of the proposed vaccine allocation strategy will be difficult to guarantee, i.e., there may be other better allocation strategies (see the Supplementary Materials). The effect of spatio-temporal heterogeneity of epidemiological landscapes on vaccine allocation is ignored to some extent in previous studies. These studies lack the optimal deployment of vaccines under different epidemiological landscapes.

The current vaccine spatial allocation strategy usually allocates available doses to sub-regions in proportion to the size of their population (referred to as the pro-rata allocation). For policymakers, this strategy may be easier to implement. However, such a strategy that ignores epidemiological landscapes may not always maximize the population effectiveness of vaccines. In this view, we proposed a new model of vaccination allocation using a graph-based spatial model and an extended SIR-based temporal model. We simulated the epidemic dynamics in subregions during vaccine deployment sufficiently through this model to set up a variety of possible vaccination scenarios. The optimization algorithm was then run under different scenarios to independently minimize two metrics of infection and disease burden: proportion of cumulative infections and deaths prevented. We also evaluated vaccine allocation in combination with non-pharmacological interventions. In epidemiology, a non-pharmaceutical intervention (NPI) is any method to reduce the spread of an epidemic disease without requiring pharmaceutical drug treatments. Vaccination is a pharmaceutical intervention by definition. However, we believe that vaccination is often not administered alone, but in conjunction with other non-pharmacological interventions. The non-pharmaceutical interventions may affect the effectiveness of vaccination strategies. There, we introduced vaccination at different intensities of non-pharmaceutical interventions to investigate the variation patterns of optimal strategies in the context of different degrees of viral transmission.

## Methods

### Vaccination Allocation Model

The vaccine allocation model consists of two components: an age-structured deterministic compartmental model to describe the temporal dynamics of infectious diseases within the sub-region and a graph-based spatial model to simulate the epidemiological landscapes that the region may face at the time of vaccination.

#### Age-Structured Deterministic Compartmental Model

We stratified individuals by 10-year age groups, in line with previous parameter estimation and data. We assumed subjects started susceptible to infection (S) and could become exposed but not yet infectious (E) after effective contact. After a latent period, exposed individuals developed an asymptomatic infection (A) or a symptomatic infection (I). Both symptomatic and asymptomatic infectious individuals had a certain probability of being detected (ID). Infectious individuals eventually recovered (R) or died (D) depending on the severity of their symptoms. Among them, asymptomatic individuals did not die but recovered at a given rate. We also assumed that recovered individuals became susceptible again at a given rate, reflecting eventual loss of temporary immunity from the infection (30, 31). We used the previously estimated (32) contact matrix C with age structure and corrected for reciprocity (33) based on the existing demographic structure of the subregion (34), adapting it to each subregion.

We introduced a leaked vaccine into the model and tracked the vaccinated individuals by subscript *v*. Vaccination was available to susceptible and recovered individuals. We assumed that the vaccine can have two effects on the vaccinated individuals. First, they are less likely to be infected with the virus compared to unvaccinated individuals (this effect is expressed by *V*_*e*_). Second, the vaccine can also reduce the probability of developing symptoms upon infection (referred to as *V*_*p*_). Vaccine effect on COVID-19 disease is defined as reduction in the likelihood to develop symptomatic diseases upon exposure (referred to as *V*_*dis*_). The relationship (35) among *V*_*dis*_, *V*_*e*_, and *V*_*p*_ is shown as follows:


(1) $$\begin{array}{l} V_{dis}=1-\left(\left(1-V_{e}\right)\left(1-V_{p}\right)\right) \end{array}$$

**Figure 1:**
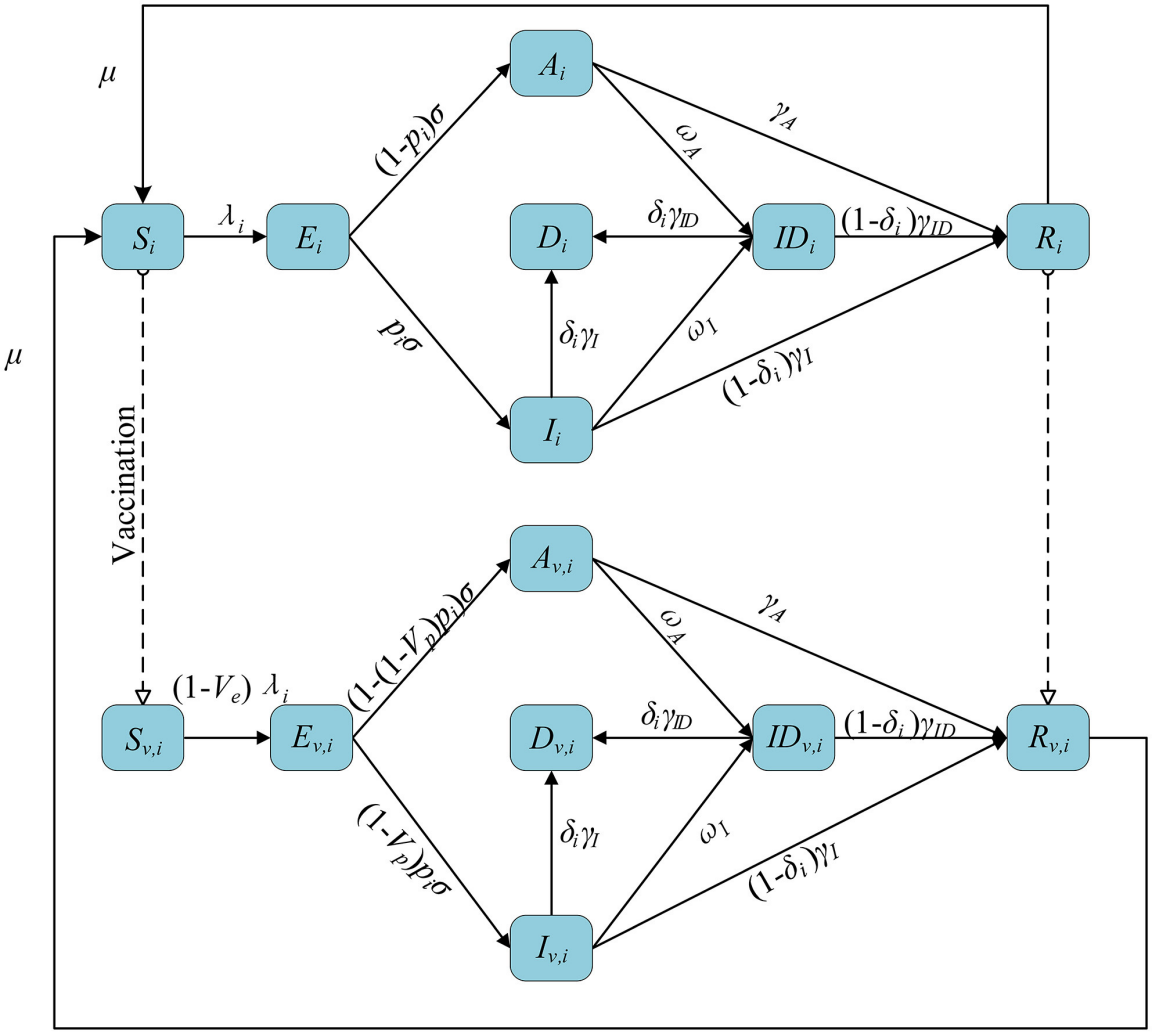
The flowchart of the transmission model.

A flowchart of the model is presented in Figure 1. The equations used in the model are as follows:


(2) $$\begin{array}{l} c_{ij}=w_{h}c_{ij|\mathrm{hom}e}+w_{w}c_{ij|work}+w_{s}c_{ij|school}+w_{o}c_{ij|other} \end{array}$$

(3) $$\begin{array}{l} \lambda_{i}=\beta m_{i}\overset{9}{\underset{j=1}{\sum }}c_{ij}\left(\partial \left(A_{j}+{\text{A}}_{v,j}\right)+I_{j}+I_{v,j}\right)/N_{j} \end{array}$$

(4) $$\begin{array}{l} \frac{dS_{i}}{dt}=-\lambda_{i}S_{i}+\mu \left(R_{i}+R_{v,i}\right) \end{array}$$

(5) $$\begin{array}{l} \frac{dE_{i}}{dt}=\lambda_{i}S_{i}-\sigma E_{i} \end{array}$$

(6) $$\begin{array}{l} \frac{dA_{i}}{dt}=\left(1-p_{i}\right)\sigma E_{i}-\left(\gamma_{A}+\omega_{A}\right)A_{i} \end{array}$$

(7) $$\begin{array}{l} \frac{dI_{i}}{dt}=p_{i}\sigma E_{i}-\left(\gamma_{I}+\omega_{I}\right)I_{i} \end{array}$$

(8) $$\begin{array}{l} \frac{dID_{i}}{dt}=\omega_{A}A_{i}+\omega_{I}I_{i}-\gamma_{ID}ID_{i} \end{array}$$

(9) $$\begin{array}{l} \frac{dR_{i}}{dt}=\gamma_{A}A_{i}+\left(1-\delta_{i}\right)\left(\gamma_{I}I_{i}+\gamma_{ID}ID_{i}\right)-\mu R_{i} \end{array}$$

(10) $$\begin{array}{l} \frac{dD_{i}}{dt}=\delta_{i}\left(\gamma_{I}I_{i}+\gamma_{ID}ID_{i}\right) \end{array}$$

(11) $$\begin{array}{l} \frac{dS_{v,i}}{dt}=-\left(1-V_{e}\right)\lambda_{i}S_{v,i} \end{array}$$

(12) $$\begin{array}{l} \frac{dE_{v,i}}{dt}=\left(1-V_{e}\right)\lambda_{i}S_{v,i}-\sigma E_{v,i} \end{array}$$

(13) $$\begin{array}{l} \frac{dA_{v,i}}{dt}=\left(1-\left(1-V_{p}\right)p_{i}\right)\sigma E_{v,i}-\left(\gamma_{A}+\omega_{A}\right)A_{v,i} \end{array}$$

(14) $$\begin{array}{l} \frac{dI_{v,i}}{dt}=\left(1-V_{p}\right)p_{i}\sigma E_{v,i}-\left(\gamma_{I}+\omega_{I}\right)I_{v,i} \end{array}$$

(15) $$\begin{array}{l} \frac{dID_{v,i}}{dt}=\omega_{A}A_{v,i}+\omega_{I}I_{v,i}-\gamma_{ID}ID_{v,i} \end{array}$$

(16) $$\begin{array}{l} \frac{dR_{v,i}}{dt}=\gamma_{A}A_{v,i}+\left(1-\delta_{i}\right)\left(\gamma_{I}I_{v,i}+\gamma_{ID}ID_{v,i}\right)-\mu R_{v,i} \end{array}$$

(17) $$\begin{array}{l} \frac{dD_{v,i}}{dt}=\delta_{i}\left(\gamma_{I}I_{v,i}+\gamma_{ID}ID_{v,i}\right) \end{array}$$

where *c*_*ij*_ is the number of individuals in age group *j* contacted by an individual in age group *i*, *c*_*ij*|hom*e*_, *c*_*ij*|*work*_, *c*_*ij*|*school*_, and *c*_*ij*|*other*_ are the number of contacts at home, work, school, and other locations, respectively, and *w*_*h*_, *w*_*w*_, *w*_*s*_, and *w*_*o*_ are the weight of the contact matrix of the four locations, respectively. λ_*i*_ is the force of infection for an individual in age group *i*, β is the transmission coefficient, μ_*i*_ is the relative susceptibility to infection for age-*i*, and α is the relative infectiousness of asymptomatic infections. 1/μ is the average length of immunity. 1/σ is the mean duration of latent period. *p*_*i*_ is the proportion of the symptomatic in age group *i*. 1/γ_*A*_, 1/γ_*I*_, and 1/γ_*ID*_ are the average recover time of the asymptomatic, symptomatic and detected, respectively. ω_*A*_ and ω_*I*_ are the detection rate (*via* contact tracing and testing) for asymptomatic and symptomatic infections, respectively. δ_*i*_ is the fatality of the disease for age-*i* individuals.

#### Graph-Based Spatial Model

**Figure 2:**
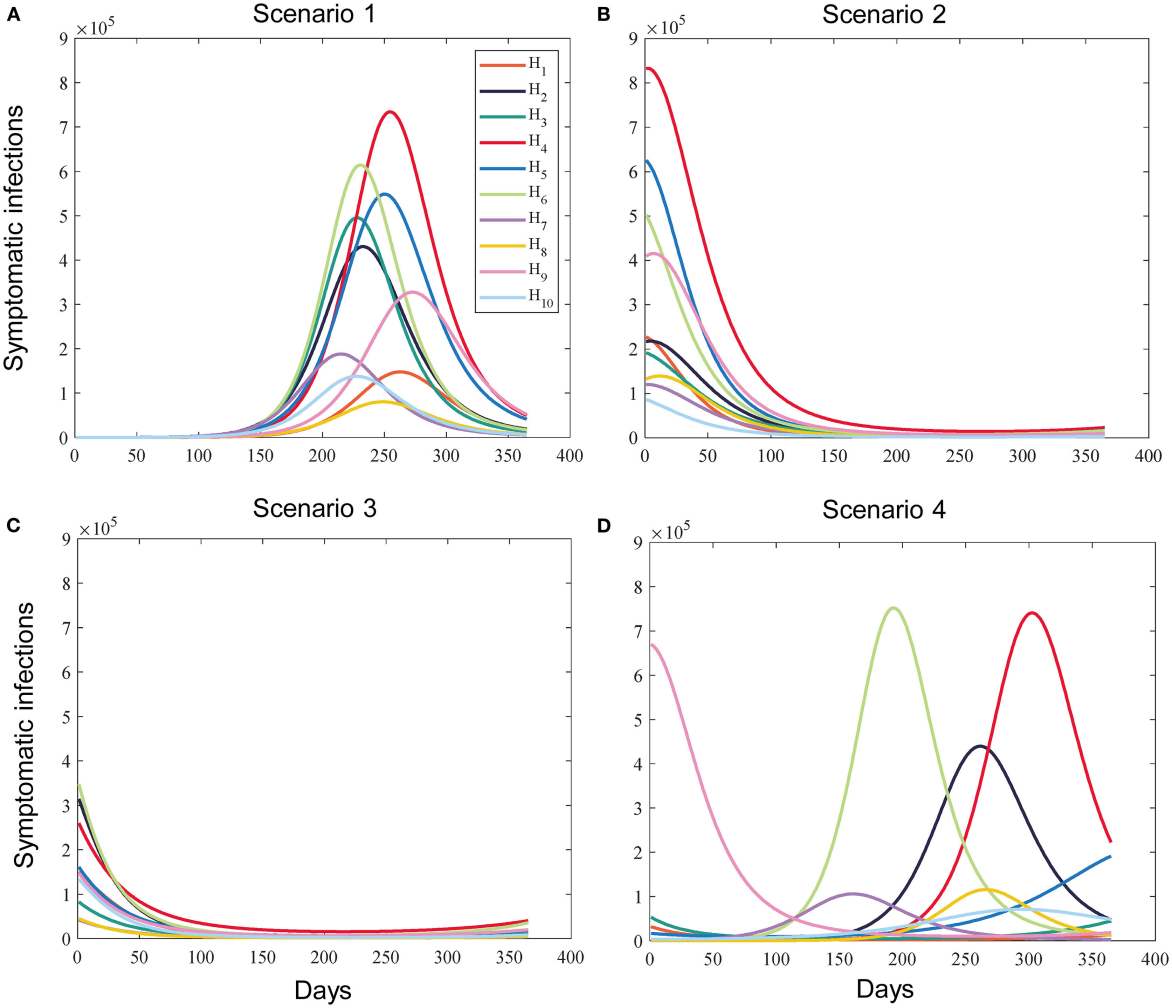
Example of the simulated prevalence of sub-regional symptomatic infections under different scenarios without vaccination. **(A)** Scenario 1 (All subregions are in the early stages of the pandemic). **(B)** Scenario 2 (All subregions are in the middle stages of the pandemic). **(C)** Scenario 3 (All subregions are in the late stages of the pandemic). **(D)** Scenario 4 (The epidemic dynamics of the subregions differ significantly).

Due to the spatio-temporal heterogeneity and uncertainty in the spread of infectious diseases and the implementation of interventions, the epidemiological dynamics of sub-regions when deploying vaccines can be either similar (same early, middle or late stage of a pandemic) or very different (sub-regions at different times of the pandemic). We introduced into the model the times at which vaccination begins in sub-regions to set up a series of possible vaccination scenarios based on this phenomenon. Different scenarios represent different epidemiological landscapes, as shown in Figure 2. For sub-region *k* under scenario s, if vaccination starts on day $t_{k}^{s}$, it means that the virus has been transmitted for ($t_{k}^{s}$ – 1) days. Depending on the scenario, the times were randomly generated within a reasonable range. This will affect the initial status of each compartment, such as sub-regions that are in the early stage of the pandemic with a greater proportion of susceptible individuals. We quantified the intensity of the intervention using the control reproduction number (*R*_*c*_, defined as the average number of secondary COVID-19 infections produced by typically infected individuals in a susceptible population with control measures). The *R*_*c*_ values for sub-regions were also randomly generated within a certain range. We used these *R*_*c*_ values to estimate some of the parameters in the model that were relevant to non-pharmacological interventions. This means that these parameters may be different for different sub-regions. Full simulation details can be found in subsection Vaccination Simulation Scenarios.

We considered a baseline basic reproductive number *R*_0_ = 2.5 (no interventions) to calculate the transmission coefficient β. We defined the next-generation matrix (NGM) as


(18) $$\begin{array}{l} NGM_{ij}=\beta m_{i}\underset{j}{\sum }c_{ij}\left(\partial d_{A}+d_{I}\right) \end{array}$$

*R*_0_ is the spectral radius of the NGM (36). *d*_*A*_and *d*_*I*_ denote respectively the length of time individuals spend in states *A* and *I*.

### Risk Assessment of Viral Transmission in Subregions

The effective reproduction number (*R*_*e*_, defined as the average number of secondary cases per infectious case in a population made up of both susceptible and non-susceptible hosts) was usually used to measure epidemic transmission. Its value will change as the pandemic proceeds, as some people may have gained immunity through infection or vaccination. It is not very desirable to use the effective reproduction number at a given moment in isolation when prioritizing resources. Policymakers need to look at the whole picture and use it alongside other indicators. As shown in Equation 19, we assessed the risk of transmission in subregions by the effective reproduction number and the prevalence of symptomatic infections.


(19) $$\begin{array}{l} {\text{r}}_{k}=RS_{k}+\eta IS_{k} \end{array}$$

where r_*k*_ is the risk of viral transmission in the sub-region *k*. *RS*_*k*_ and *IS*_*k*_ are the total effective reproduction number (sum of effective reproduction number over *T* (our time horizon) days) and the total prevalence of symptomatic infections (sum of symptomatic infections over *T* days) without vaccination in the sub-region *k*, respectively, both of which are normalized. Considering that *R*_*e*_ could better reflect the transmission risk, we assumed η = 0.5.

### Optimization Algorithm

The main focus of our study was on time and space. Within the subregion, our optimizer would prioritize vaccine allocation to the elderly. This is in line with most current policies and is more ethical. Two objective functions were ultimately chosen: the proportion of cumulative symptomatic infections and deaths prevented 1 year (our time horizon) after vaccination initiation compared with the unvaccinated base case. The reason why we choose symptomatic infections is that these individuals are more infectious compared to asymptomatic infections, and minimizing symptomatic infections is better for controlling the further spread of the virus. Moreover, symptomatic infections are closely related to public health policy concern: the burden on the health system. Therefore, we believe that although policies tailored to reduce deaths are of great significance, there is still a need to optimize the allocation of vaccines for symptomatic infections. This can provide more insight and flexibility in the formulation of public health policy.


(20) $$\begin{array}{l} \underset{X}{\min}f\left(X\right) \end{array}$$

(21) $$\begin{array}{l} s.t.\{\begin{array}{c} \;\underset{k}{\sum }X_{k}=1\\ \;X_{k}N\leq P_{k}\\ \;0\leq X_{k}\leq 1 \end{array} \end{array}$$

where *f* (*X*) is the objective function and *X* is the decision variable. *X*_*k*_ is the proportion of vaccines allocated to subregion *k* to the total available vaccines. *P*_*k*_ is the number of people in sub-region *k* and *N* is the total number of people in the region.

To increase the chances of approaching the global optimal solution, we combined coarse global search with genetic algorithm to explore the entire space of possible combinations of vaccine allocation. Before running the genetic algorithm, we performed coarse-grained grid search over the entire decision variable space, with a search step of 0.1. For example, a point in the grid is *X* = (0,0.1,0,0,0.1,0.4,0.2,0.2,0,0), which represents the sub-regions 2, 5, 6, 7, 8 are allocated to 10, 10, 40, 20, and 20% of the available vaccines, respectively. Each grid point needed to satisfy the above constraints, if not, the grid point would be discarded. We evaluated the objective function on all feasible grid points and selected the best 25 points. The best 25 points obtained above and the pro-rata allocation vector were put into the initial population of the genetic algorithm, and the remaining individuals of the initial population were randomly generated by the genetic algorithm according to the constraints. It is worth noting that under high vaccine supply (60−100%), the selection of feasible grid points may be <25 due to the reduction in the number of decision variables that meet the constraints. If the vaccine supply reaches 100%, the only theoretically feasible decision variable is the pro-rata vector. In this case, all individuals of the initial population were randomly generated by the algorithm itself.

## Results

We partitioned the continental US into the 10 Standard Federal Regions (10 HHS Regions) established by the US Office of Management and Budget. The 10 HHS Regions are our study subjects. More information on the 10 HHS regions can be found in Supplementary Materials. Given the current *V*_*dis*_ data and the uncertainty in the specific *V*_*e*_ and *V*_*p*_ values, we evaluated the optimal use of 50 vaccines with specific effectiveness and coverage (*V*_*dis*_ ranging from 60 to 100% and vaccination coverage ranging from 10 to 100% of the total population, in in-crements of 10%). We set *V*_*e*_ = 50% while varying the value of *V*_*p*_ according to *V*_*dis*_.

Our study included four categories of scenarios and three intensities of non-pharmacological interventions. We assumed that the intensities of the non-pharmacological interventions in subregions were close and set, [2.0,2.3], [1.5,1.8], and [1.2,1.4] for low, moderate, and high intensity non-pharmacological interventions, respectively. We assumed that sub-regions deployed vaccines at a certain rate per day (rollout speeds of 0.05% of their population per day) until the supply was exhausted. Since each country will have different vaccination rates, the at-once allocation would make the results more general. Therefore, we also used this method to determine the near-optimal strategy under different scenarios (see the Supplementary Materials). All simulations were performed on the Matlab 2021a platform. For the genetic algorithm, we used the global optimization toolbox provided by it. The implementation details and hyperparameters of the genetic algorithm are shown in Table 1.

**Table 1:** The values for the hyperparameters of the GA.

**Hyperparameter**	**Value**
Population size	350
Elite count	18
Crossover fraction	0.8
Mutation fraction	0.15
Stall generations^*^	250
Function tolerance^*^	10^−6^

* *The algorithm stops if the average relative change in the best fitness function value over MaxStallGenerations is less than or equal to FunctionTolerance*.

### Simulation Setup

#### Parameter Estimation

The values of most parameters in the model referred to previous studies, while some of the parameters related to non-pharmacological interventions were estimated using genetic algorithm. The decision variables of the genetic algorithm are all parameters to be estimated. The fitness value is the absolute value of the difference between the target *R*_*c*_ and the estimated *R*_*c*_. The values of relevant parameters are shown in Table 2.

**Table 2:** The values and source of parameters in the model.

**Parameter**	**Value**	**Method**
1/σ	6	(34)
1/γ_*A*_	7	(3)
1/γ_*I*_	7	(3)
1/γ_*ID*_	15	(3)
α	0.5	(3)
ω_*A*_	–	Estimated
ω_*I*_	–	Estimated
1/μ	365	Assumed
β	–	Calculated
*m*_*i*_	[0.4, 0.38, 0.79, 0.86, 0.8, 0.82, 0.88, 0.74, 0.74]	(37)
*p*_*i*_	[0.29, 0.21, 0.27, 0.33, 0.4, 0.49, 0.63, 0.69, 0.69]	(37)
δ_*i*_	[0.00002, 0.00006, 0.00031, 0.0008 0.0015, 0.006, 0.022, 0.051, 0.093]	(38)
*w*_*h*_, *w*_*w*_, *w*_*s*_, *w*_*o*_	–	Estimated
*c*_*ij*|hom*e*_, *c*_*ij*|*work*_, *c*_*ij*|*school*_, *c*_*ij*|*other*_	–	(32)

#### Vaccination Simulation Scenarios

The possible scenarios were divided into two main categories: epidemic dynamics in sub-regions are relatively close or different significantly. For the first category of scenarios, we built three sub-scenarios (Scenarios 1, 2, and 3). Scenarios 1, 2 and 3 represent epidemiological landscapes with all subregions in the early, middle, and late stages of the pandemic, respectively, while scenario 4 represents that the epidemiological dynamics of the sub-regions are significantly different. The generation intervals of the times when sub-regions start vaccination under different scenarios are as follows:


(22) $$\begin{array}{lll} t_{k}^{s} & \in & \{\begin{array}{l} \;\left(p_{a}t_{k}^{p},\left(p_{a}+l_{w}\right)t_{k}^{p}\right),\;s=1\\ \;\left(\left(1-\frac{1}{2}l_{w}\right)t_{k}^{p},\left(1+\frac{1}{2}l_{w}\right)t_{k}^{p}\right),\;s=2\\ \;\left(\left(1+p_{a}\right)t_{k}^{p},\left(1+p_{a}+l_{w}\right)t_{k}^{p}\right),\;s=3\\ \;\left(0,t_{k}^{l}\right),\;s=4 \end{array} \end{array}$$

(23) $$\begin{array}{l} l_{w}=\{\begin{array}{ll} 0.1, & s=1,2,3\\ 1, & s=4 \end{array} \end{array}$$

where $t_{k}^{s}$ represents the time when vaccination begins in sub-region *k* under scenario *s*. $t_{k}^{p}$ represents the expected timing of the pandemic peak in sub-region *k*. *l*_*w*_ is the scaling factor of the generation interval to control the degree of proximity of the epidemic dynamics among sub-regions. *p*_*a*_ (0 < *p*_*a*_ < 1–*l*_*w*_) is used to determine the location of the interval. In scenarios 1 and 3, we adjusted the value of *p*_*a*_ and conducted multiple experiments to check whether the near-optimal vaccine strategy changed when the interval changed. $t_{k}^{l}$ is the pandemic duration for sub-region *k*.

### Uncertainty and Sensitivity Analysis of Model Parameters

**Figure 3:**
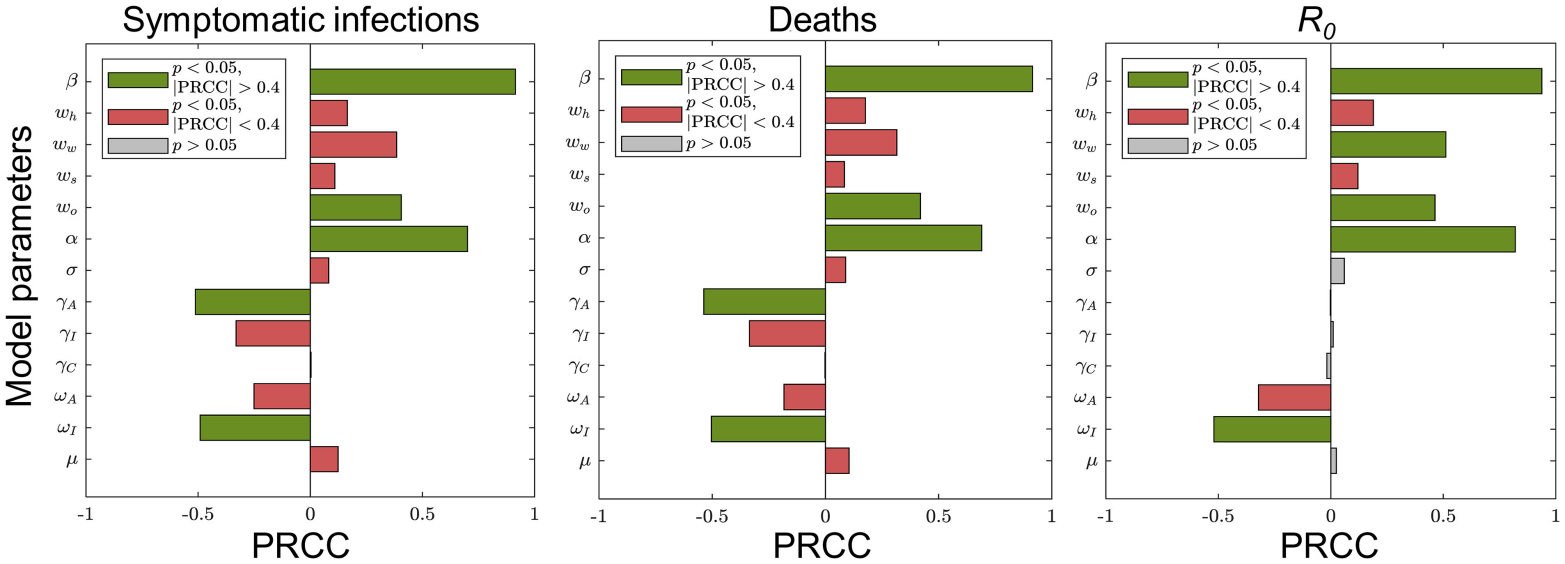
Sensitivity analysis of the main parameters of the model. Each plot shows the sensitivity of the corresponding response function value to changes in a single main input parameter of the model. The green (red) bars indicate sensitive (insensitive) and gray bars indicate results that are not statistically significant.

We assumed that each parameter obeys a uniform distribution over its range of values. Latin hypercube sampling (LHS) was performed for the entire parameter space. The model was run 1,000 times to analyze the sensitivity of response function values to changes in a single input parameter using the partial rank correlation coefficient (PRCC) as an indicator. The results are shown in Figure 3.

These results suggest that COVID-19 can be controlled effectively in the population by some interventions such as wearing masks, maintaining social distance, or large-scale vaccination. We chose model parameters that were more sensitive to the objective functions as parameters to be estimated: weights of contacts at home, school and other locations, and detection rates for asymptomatic as well as symptomatic infections.

### Near-Optimal Vaccine Allocation Differs for Different Epidemiological Landscapes

The near-optimal spatio-temporal allocation for the same scenario is nearly identical under different intensities of non-pharmacological interventions. In this section, we analyzed how the near-optimal strategy to minimize symptomatic infections changed for different epidemiological landscapes and present results under low-intensity non-pharmacological interventions.

**Figure 4:**
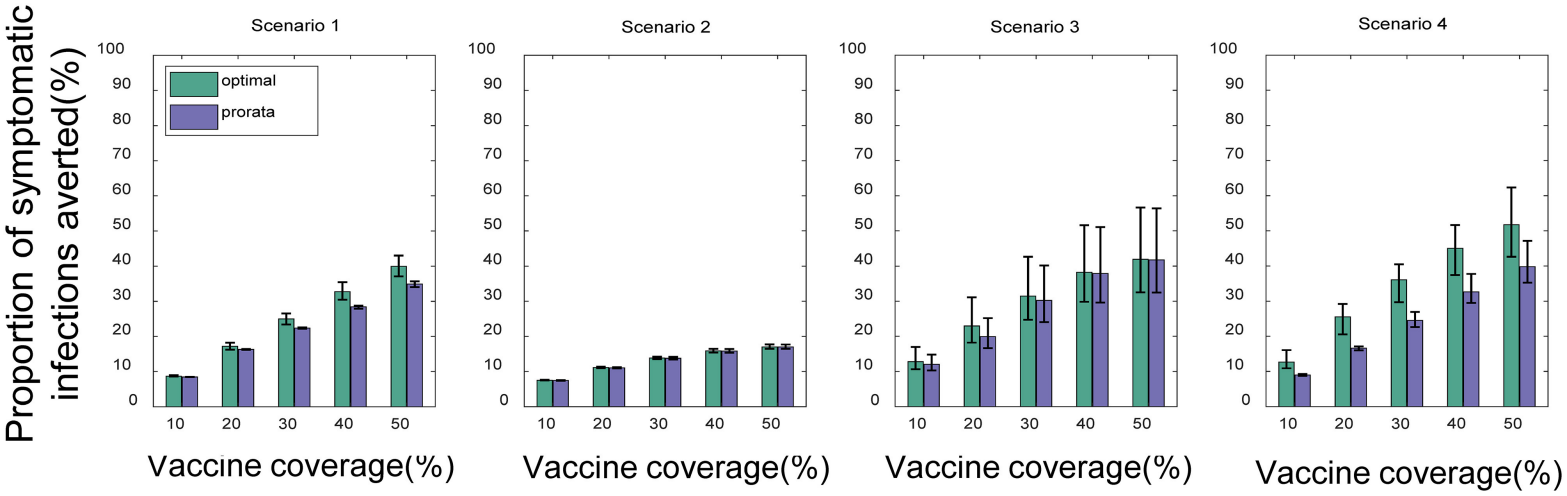
Percentage of cumulative symptomatic infections averted for both strategies under different scenarios. When *V*_*dis*_ = 60% and low-intensity non-pharmacological intervention, the percentage of cumulative symptomatic infections averted for the near-optimal strategy (green) and the pro-rata strategy (purple). Bars represent the mean of multiple experimental results and error bars represent uncertainty intervals (UI).

**Figure 5:**
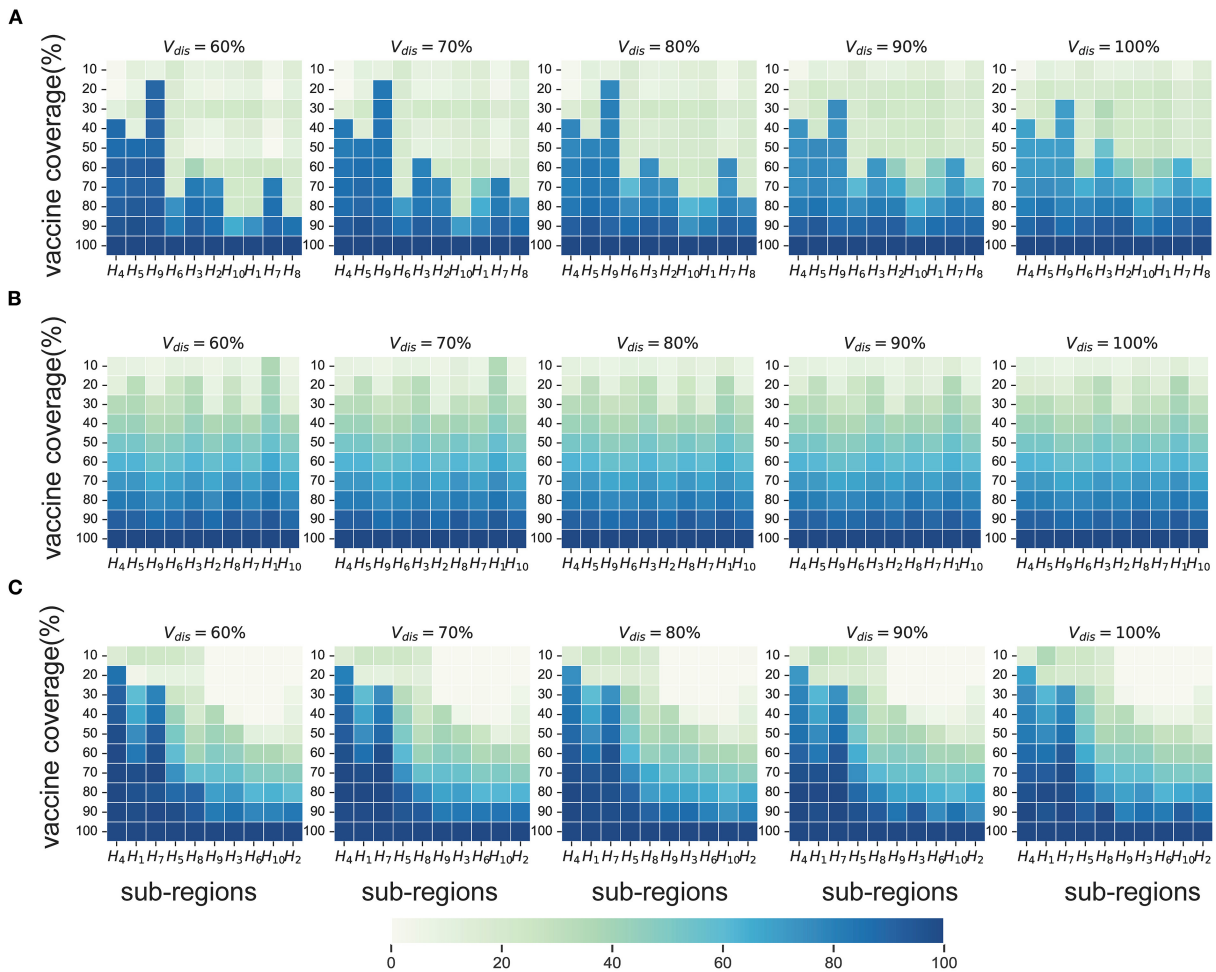
Near-optimal allocation strategies to minimize symptomatic infections under scenario 1 **(A)**, 3 **(B)** and 4 **(C)**. For each heat map, each row from left to right is the decreasing direction of transmission risk, representing the total vaccine supply (percentage of the total population vaccinated) and each column represents a different subregion. Colors represent the percentage of the population in a sub-region to be vaccinated.

Compared with no vaccination, both strategies substantially mitigated viral transmission in all scenarios, especially when vaccine coverage is relatively high. If *V*_*dis*_= 60% and vaccine coverage was 50%, the near-optimal strategy could avert 40% (UI: 37−43%), 17% (UI: 16−17%), 42% (UI: 33−57%), and 52% (UI: 43−62%) of symptomatic infections in the four scenarios on average, respectively (Figure 4). This suggests that mass vaccination can go a long way in alleviating the spread of COVID-19 and also emphasizes the importance of distributing vaccines as soon as possible. Because the population effectiveness of vaccination will diminish as the pandemic proceeds. The near-optimal strategy under scenarios 1 and 4 could provide more gain compared to other scenarios, averting up to 9% more (*V*_*dis*_ = 60%, ~60% of vaccine coverage) and 15% more symptomatic infections (*V*_*dis*_ = 60%, ~40% of vaccine coverage) at the greatest difference between the two strategies, respectively. In scenario 1, when the vaccine coverage was ~30−70%, it was optimal to vaccinate higher-risk sub-regions at high coverage, while the pro-rata allocation was close to the near-optimal strategy for other coverages (Figure 5A). In stark contrast, in Scenarios 2 and 3, the pro-rata strategy was close to optimal regardless of vaccine coverage and effectiveness (Figure 5B). The near-optimal vaccine allocation is similar for scenarios 4 and 1, with the difference that the priority was also given to higher-risk sub-regions when vaccine coverage is less than 30% (Figure 5C). This may be since that in scenario 1, the risk of transmission in the sub-regions is not very different and the virus is rapidly spreading. All subregions require large amounts of vaccines to slow the further spread of the virus. While in scenario 4, some sub-regions are already in the middle and late stages of the pandemic. In the case of insufficient vaccines, the available doses can be allocated to the sub-regions in the early stages of the pandemic. Both strategies tended to perform similarly as vaccine effectiveness and coverage increased.

### Near-Optimal Vaccine Allocation Changes With Objective Functions

Next, we investigated the effect of the vaccination objectives on the near-optimal strategy and present some of the results under moderate intensity non-pharmacological interventions. The near-optimal vaccine allocation for the two objective functions differed more in scenarios 1 and 4. While in scenarios 2 and 3, whatever the objective function was, the pro-rata allocation was close to optimal in most cases.

**Figure 6:**
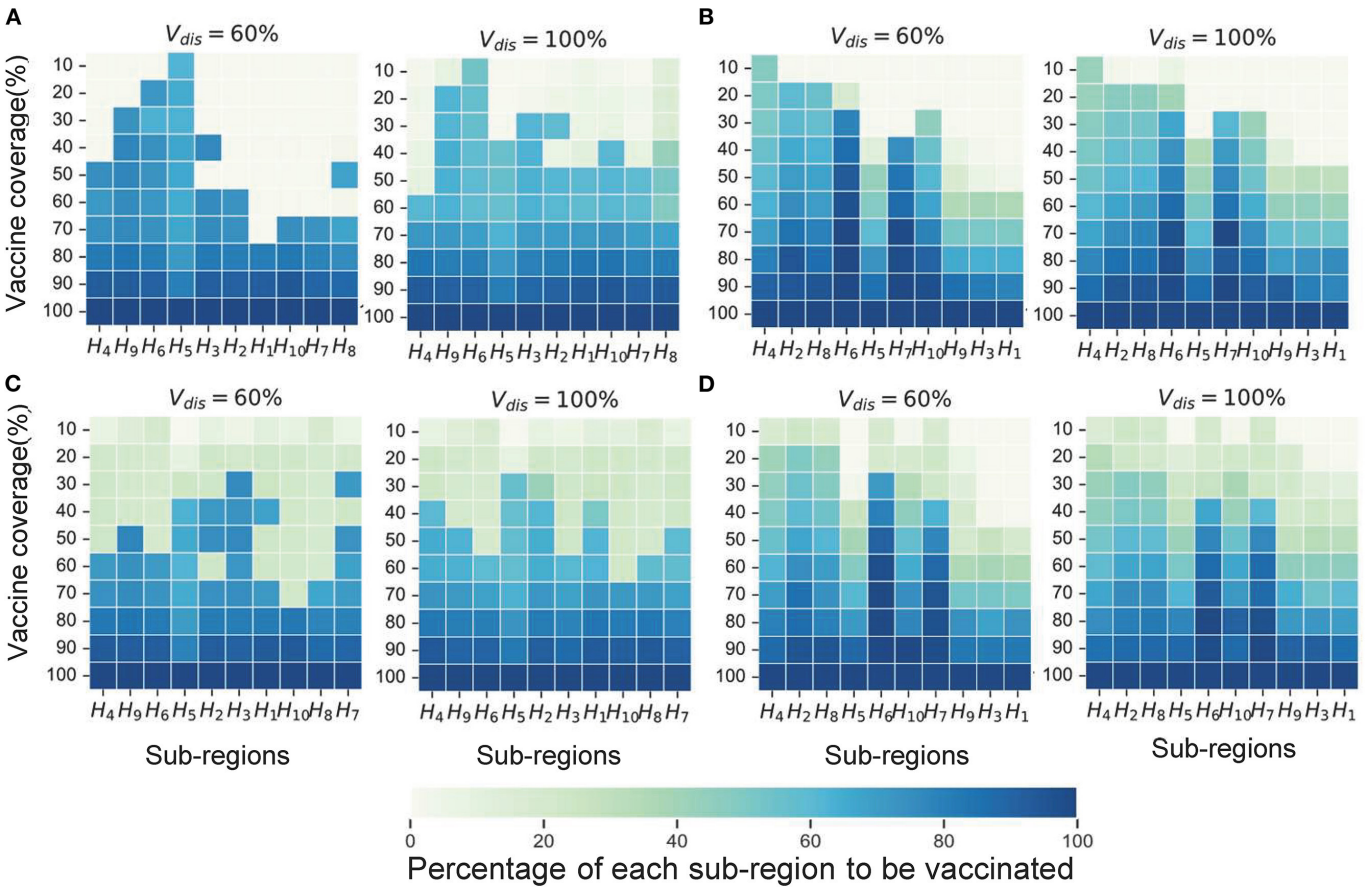
Near-optimal allocation strategies for different objective functions. Here, we only show the results for *V*_*dis*_ = 60 and 80%. **(A)** The near-optimal strategies to minimize symptomatic infections in scenario 1. **(B)** The near-optimal strategies to minimize symptomatic infections in scenario 4. **(C)** The near-optimal strategies to minimize deaths in scenario 1. **(D)** The near-optimal strategies to minimize deaths in scenario 4. For each heat map, each row from left to right is the decreasing direction of transmission risk, representing the total vaccine supply (percentage of the total population vaccinated) and each column represents a different subregion. Colors represent the percentage of the population in a sub-region to be vaccinated.

**Figure 7:**
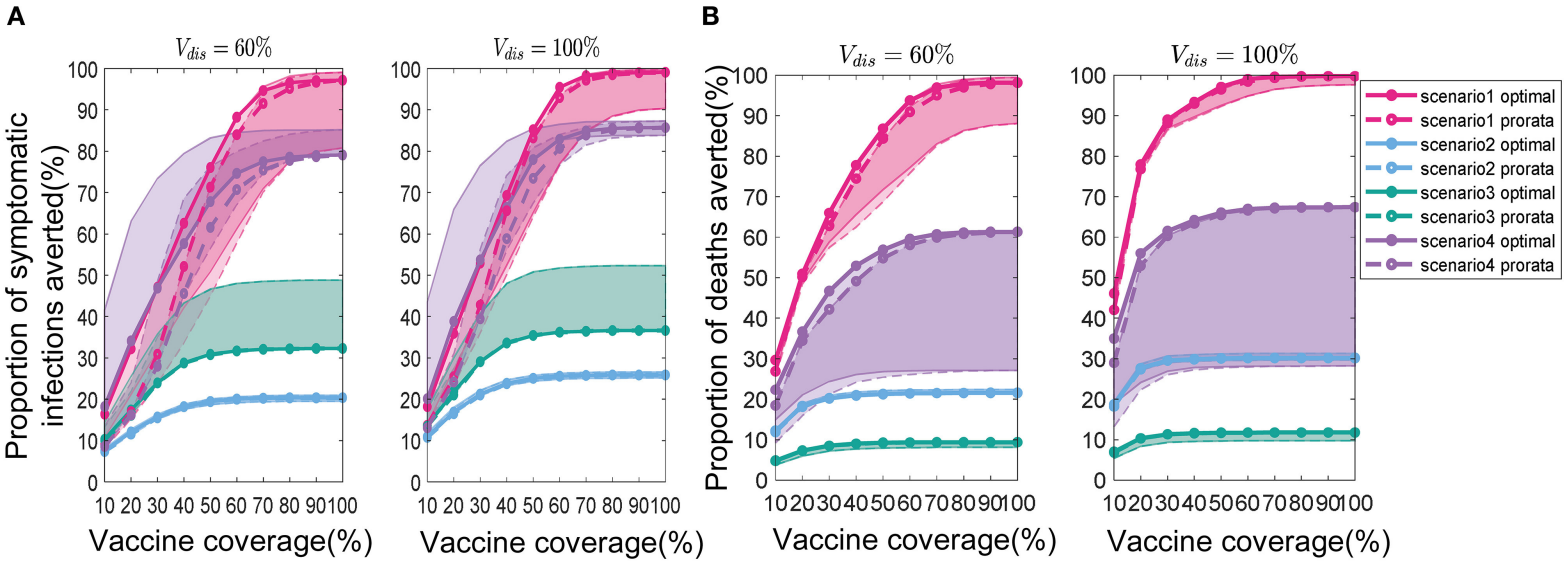
Percentage of cumulative symptomatic infections **(A)** and deaths **(B)** averted for both strategies. Solid lines with solid circles and dashed lines with hollow circles represent the near-optimal and pro-rata strategies, respectively. For clarity, we highlight 1 set of results in Figure 6 in each scenario. The shaded areas represent the range of effectiveness of strategies under multiple epidemiological landscapes simulated.

In Scenario 1, for low vaccine coverage, the near-optimal strategy to minimize symptomatic infections prioritized the coverage in higher-risk sub-regions; while when minimizing deaths, the optimizer's allocation results were similar to the pro-rata strategy. As more vaccines became available (30−60% vaccine coverage), the near-optimal strategy for symptomatic infections remained the same, while the near-optimal strategy for minimizing deaths first covered the elderly in all sub-regions as much as possible and then allocated the remaining dose to the subregion with the expected higher risk and coverage (Figures 6A,C). The main reason for this phenomenon is that the main contributors to the spread of the virus in this COVID-19 pandemic are the younger age groups, but the mortality rate in the elderly is much higher than in other age groups. In our optimizer, the vaccine is first distributed to the elderly in the sub-region. Allocating vaccine to older people in the region first helps to minimize deaths. The effective control of the epidemic requires mass vaccination among young people. The near-optimal spatio-temporal strategy under scenario 4 is overall similar to that of scenario 1, except that with fewer vaccines available, the optimization algorithm may only focus on allocating vaccines to sub-regions in the early and middle stages of the pandemic (Figures 6B,D). In addition, we found that the gain from optimizing vaccine allocation to improve pro-rata strategy was relatively small when minimizing the deaths (Figure 7). This may be because prioritizing older adults is more beneficial for minimizing deaths within a sub-region than other vaccine prioritization strategies.

### Effect of the Intensity of Non-pharmacological Interventions on Vaccine Allocation

Since the epidemic dynamics of the subregions in each experiment were randomly generated, the epidemiological landscapes under scenario 4 may vary considerably under different intervention intensities. Therefore, we only discuss the effect of the intensity of non-pharmacological interventions on the near-optimal vaccine allocation for the first 3 scenarios.

**Figure 8:**
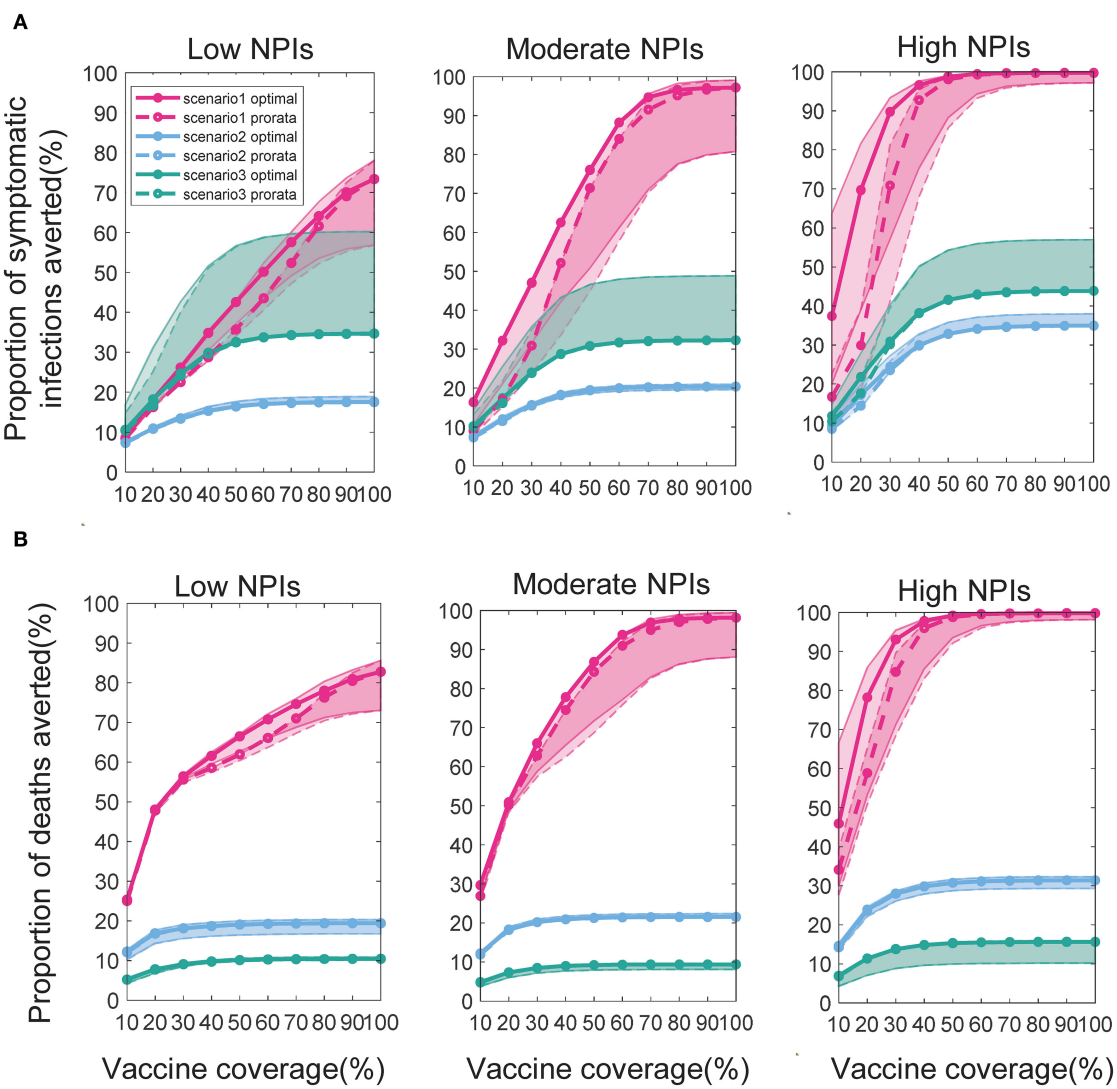
Percentage of cumulative symptomatic infections **(A)** and deaths **(B)** prevented under different intensities of interventions. When *V*_*dis*_ = 60% and vaccine coverage is 10−100% of the population, the percentage of cumulative symptomatic infections and deaths prevented for the near-optimal (solid line with solid circles) and pro-rata (dashed line with hollow circles) strategy. For clarity, we highlight 1 set of results in each scenario. The shaded areas represent the range of effectiveness of strategies under multiple epidemiological landscapes simulated.

**Figure 9:**
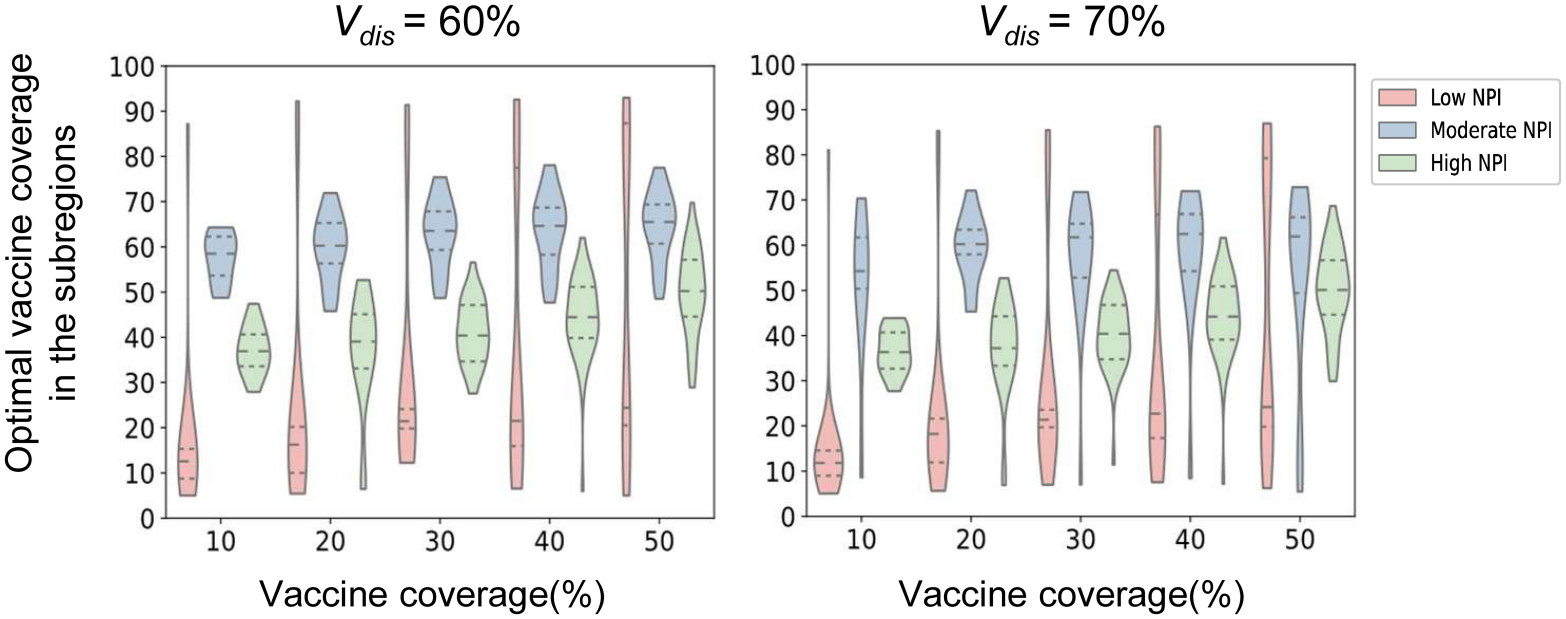
Near-optimal vaccine coverage in sub-regions. Each violin represents the distribution of the primary optimal vaccine coverage for all sub-regions in scenario 1 when minimizing the number of symptomatic infections for a certain intensity of non-pharmaceutical intervention.

We found that the intensity of non-pharmacological interventions had a greater effect on the near-optimal strategy under scenario 1. As the intensity of non-pharmacological interventions increased, the effectiveness of both strategies improved to varying degrees compared with no vaccination (Figure 8). For example, when *V*_*dis*_ = 60% and the total vaccine coverage was 50%, the near-optimal strategy in scenario 1 could prevent 43% (UI: 37−43%) of symptomatic infections if low-intensity non-pharmacological interventions were implemented along with vaccination. The symptomatic infections averted under moderate or high intensity non-pharmacological interventions would increase to 76% (UI: 51−76%) and 99% (UI: 96−99%), respectively (Figure 8A). While the effectiveness of the pro-rata strategy increased from 36% (UI: 34−36%) to 71% (UI: 45−71%) and 98% (UI: 87−99%; Figure 8A). The implementation of high-intensity non-pharmaceutical interventions not only increases the population effectiveness of vaccination but also requires fewer vaccines to control the epidemic. Vaccinating everyone in scenario 1 would still be difficult to completely control the outbreak under low-intensity non-pharmaceutical interventions. However, under high-intensity interventions, only 50% of the population would need to be near-optimally vaccinated to control the pandemic. Furthermore, when non-pharmacological interventions were intensified, the optimizer tended to allocate vaccines more evenly across all sub-regions. For the same vaccine effectiveness and coverage, the range of optimal vaccine coverage for sub-regions decreased with increasing intensity of the non-pharmacological interventions, especially when the goal of vaccination was to minimize cumulative symptomatic infections and vaccine effectiveness and coverage were not sufficiently high (Figure 9).

## Discussion

The COVID-19 pandemic is not only a serious threat to human life and health but also has a huge impact on economic development and social stability. Non-pharmacological interventions are effective in reducing the incidence of COVID-19 cases. However, in the long term, these measures are difficult to implement consistently and large-scale vaccination is highly desirable. However, during the actual deployment of the vaccine, the demand for the vaccine may far outweigh the supply. Therefore, optimizing the allocation of limited vaccines makes good practical sense and can provide valuable information for policy formulation. In this work, we proposed a novel vaccine spatio-temporal allocation model that can reproduce the epidemiological landscapes that the region may face when deploying vaccines. We used this model and optimization algorithms to determine near-optimal vaccine spatiotemporal allocation strategy in different scenarios. Our findings suggest that the performance of vaccine spatiotemporal allocation strategies is driven by several complex factors, including vaccine effectiveness and coverage, epidemic dynamics of subregions when allocating, and non-pharmacological interventions implemented.

Based on the experimental results, we provide the following recommendations for vaccination policy. If all subregions are in the middle or late stage of the pandemic when the vaccine is deployed, the existing pro-rata strategy is desirable, regardless of vaccine effectiveness and coverage. This is because under such epidemiological landscapes, for both vaccination targets, the gains from optimizing vaccine allocation are minimal compared to the pro-rata allocation. When sub-regions are in the same period of rapid virus transmission or when the epidemic dynamics of sub-regions differ significantly, prioritizing the higher-risk sub-regions may be more beneficial in controlling further virus transmission. While to minimize the deaths in the region, the elderly in all subregions need to be vaccinated as soon as possible. Under scenarios 1 and 4, the gain from optimizing vaccine allocation to improve pro-rata strategy was relatively small when minimizing the deaths. When the vaccine coverage is less than 30%, the effectiveness of the two strategies is almost the same under scenario 1. Based on this phenomenon, we suggest that older adults in all subregions should be vaccinated simultaneously if all subregions are in a period of rapid virus transmission, while older adults in subregions still in the early stages of a pandemic could be vaccinated first when the epidemiologic dynamics of each subregion differ significantly. After covering older adults in that region, priority can be given to high-risk subregions. In addition, our results suggest that the synergistic implementation of multiple pandemic interventions can lead to better pandemic control and that enhanced non-pharmaceutical interventions can reduce vaccination pressure. If non-pharmaceutical interventions are enhanced when deploying vaccines, the optimization strategy will tend to allocate the vaccine more evenly across subregions. This means that vaccines can be allocated more evenly across sub-regions based on the above recommendations to further enhance the effectiveness of vaccination if public health officials can encourage or urge the public to maintain good self-protection habits while vaccinating.

It is worth noting that this work still has certain limitations. First, the model assumed that the vaccine provided equal protection to all vaccinated individuals and that the effect was constant over the simulated time. Nevertheless, there may be some differences in the protective effect of the vaccine in different age groups, and asymptomatic infected individuals may result in weaker protection (39). The protective effect of artificial immunity induced by vaccination may decline over time (40). If immunity is transient, then these results will apply only for that duration. Second, the same rollout speed may be less desirable, so different rollout speeds for subregions may be the focus of our subsequent study, such as a faster rollout of vaccines in sub-regions with more supply.

## Data Availability Statement

The datasets presented in this study can be found in online repositories. The names of the repository/repositories and accession number(s) can be found at: https://zenodo.org/record/6574113#.You7Ke5BxPY.

## Author Contributions

WC and JZ designed research and conceived the experiments. WC, JZ, and HD conducted the experiments and analyzed the results. WC, JZ, and XW contributed to the drafting of the work. WC, JZ, XW, XT, YT, and ZM contributed to the review and editing of the manuscript. All authors read and approved the final manuscript.

## Funding

This study was funded by the National Key Research and Development Program of China (2018YFB0505304) and the National Natural Science Foundation of China (Grant No. 41671409).

## Conflict of Interest

The authors declare that the research was conducted in the absence of any commercial or financial relationships that could be construed as a potential conflict of interest.

## Publisher's Note

All claims expressed in this article are solely those of the authors and do not necessarily represent those of their affiliated organizations, or those of the publisher, the editors and the reviewers. Any product that may be evaluated in this article, or claim that may be made by its manufacturer, is not guaranteed or endorsed by the publisher.

## Abbreviations

COVID-19: coronavirus disease 2019
NPIs: non-pharmaceutical interventions
*R*_0_: basic reproduction number
*R*_*e*_: effective reproduction number
*R*_*c*_: control reproduction number
NGM: next-generation matrix.
