# Supplementary material for: Optimizing Spatio-Temporal Allocation of the COVID-19 Vaccine Under Different Epidemiological Landscapes
Source: Front Public Health. 2022 Jun 23;10:921855. doi: 10.3389/fpubh.2022.921855 (PMC9261481; doi:10.3389/fpubh.2022.921855)
Supplement: Supplementary file 1 [file Data_Sheet_1.pdf]

Supplementary Material

1 Research Subjects

The states included in each HHS region and the size of their populations are shown in the Table S1 below. Figure S1 represents the distribution of the 10 HHS regions.

Table S1 The states included in each HHS region and the size of their populations

HHS Region	States	Population size
Region 1	Connecticut, Maine, Massachusetts, New Hampshire, Rhode Island, and Vermont	14,825,551
Region 2	New Jersey, New York, Puerto Rico, and the Virgin Islands	28,301,413
Region 3	Delaware, District of Columbia, Maryland, Pennsylvania, Virginia, and West Virginia	30,689,650
Region 4	Alabama, Florida, Georgia, Kentucky, Mississippi, North Carolina, South Carolina, and Tennessee	66,572,988
Region 5	Illinois, Indiana, Michigan, Minnesota, Ohio, and Wisconsin	52,493,903
Region 6	Arkansas, Louisiana, New Mexico, Oklahoma, and Texas	42,549,343
Region 7	Iowa, Kansas, Missouri, and Nebraska	14,090,205
Region 8	Colorado, Montana, North Dakota, South Dakota, Utah, and Wyoming	12,200,880
Region 9	Arizona, California, Hawaii, Nevada, American Samoa, Commonwealth of the Northern Mariana Islands, Federated States of Micronesia, Guam, Marshall Islands, and Republic of Palau	51,055,149
Region 10	Alaska, Idaho, Oregon, and Washington	14,273,520

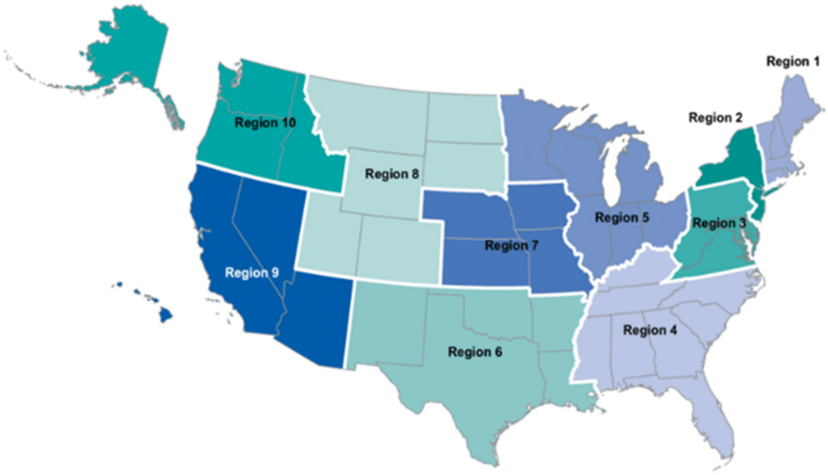

Figure S1: Distribution of the 10 HHS regions.

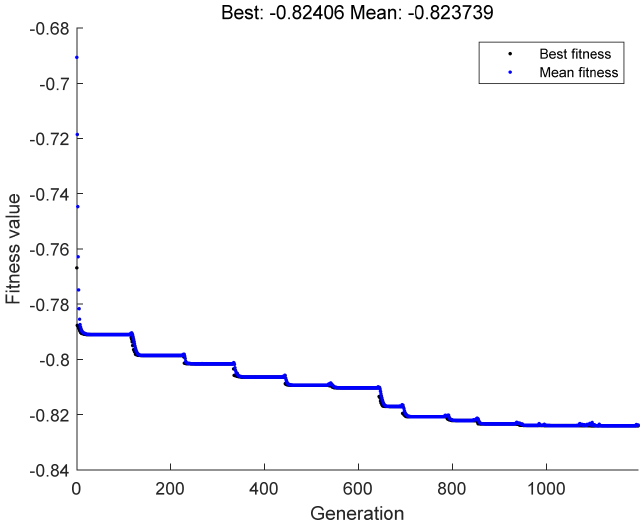

Figure S2: Example of convergence graph.

2 Effectiveness of previously proposed strategies under different epidemiological landscapes

Here, we tried to evaluate the variation in the effectiveness of previously proposed vaccine allocation strategies [22,23,26] under different epidemiological landscapes. In these strategies, sub-regions are prioritized based on certain metrics. Vaccines are allocated in order of priority, with the next sub-region considered only when the previous sub-region fulfills the target coverage (50%). Common sub-region prioritization includes: (1) peak ascending order (2) peak descending order (3) peak prevalence descending order (4) peak prevalence ascending order (5) proportion of recovered individuals in descending order (6) proportion of recovered individuals in ascending order (7) proportion of susceptible individuals in descending order (8) proportion of susceptible individuals in ascending order (9) proportion of symptomatic infections in descending order (10) proportion of symptomatic infections in ascending

order (11) pro-rata allocation. We found that the effectiveness of vaccine allocation strategies is impacted by epidemiological landscapes. Optimal vaccine allocation strategies change with epidemiological landscapes.

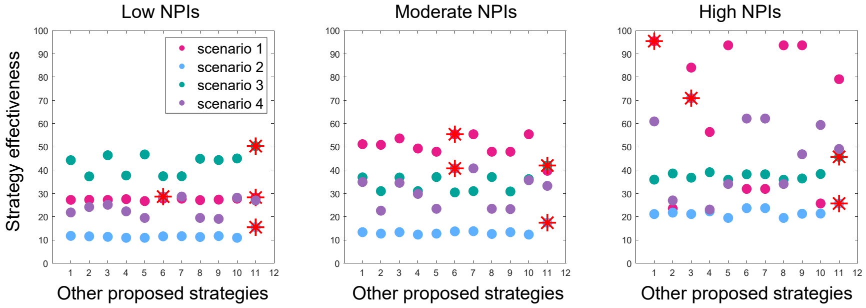

Figure S3: Effectiveness of other proposed strategies under different epidemiological landscapes. The red asterisk represents the allocation strategy that works best in each scenario.

3 At-once allocation

The total available vaccines in the model were mainly distributed at once by a given coverage. That is, we initialized the model by dividing each age group in the subregion into the susceptible compartment (S_i) and vaccination compartment ($S_{v,i}$) according to the vaccine prioritization strategy and available vaccine doses when vaccination was incorporated. We rerun all simulations under different scenarios and non-pharmaceutical control intensities. In these simulations, we only changed the vaccine allocation method. Other parameters of the model and optimization algorithm remained the same. The results of all simulations are shown below.

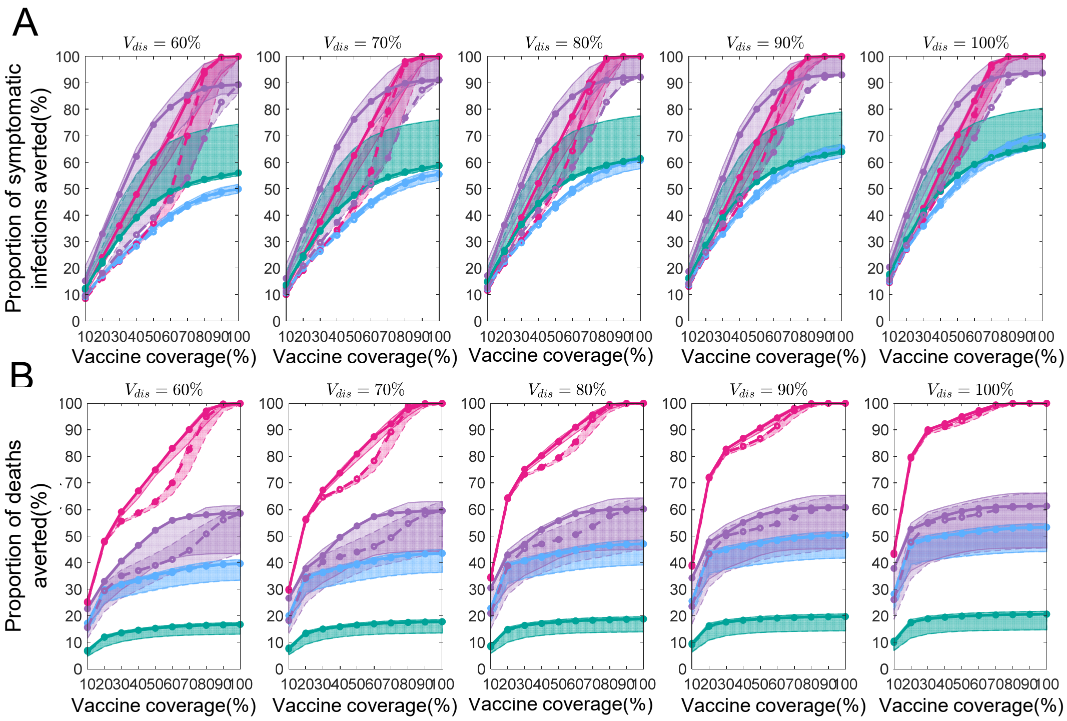

Figure S4: Percentage of symptomatic infections (A) and deaths (B) averted for both strategies under low-intensity non-pharmacological interventions. Solid lines with solid circles and dashed lines with hollow circles represent the near-optimal and pro-rata strategies, respectively. For clarity, we highlight the results of one simulation in each scenario. The shaded areas represent the results of multiple epidemiological landscape simulations. The shaded areas represent the range of effectiveness of strategies under multiple epidemiological landscapes simulated.

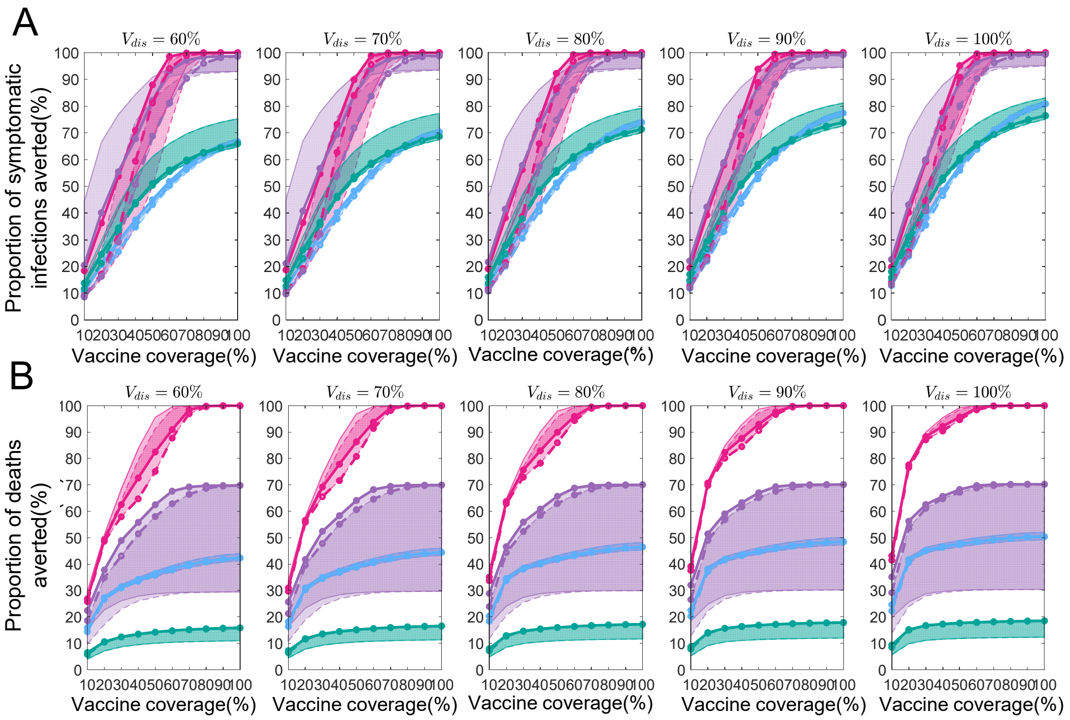

Figure S5: Percentage of symptomatic infections (A) and deaths (B) averted for both strategies under moderate-intensity non-pharmacological interventions. Solid lines with solid circles and dashed lines with hollow circles represent the near-optimal and pro-rata strategies, respectively. For clarity, we highlight the results of one simulation in each scenario. The shaded areas represent the results of multiple epidemiological landscape simulations. The shaded areas represent the range of effectiveness of strategies under multiple epidemiological landscapes simulated.

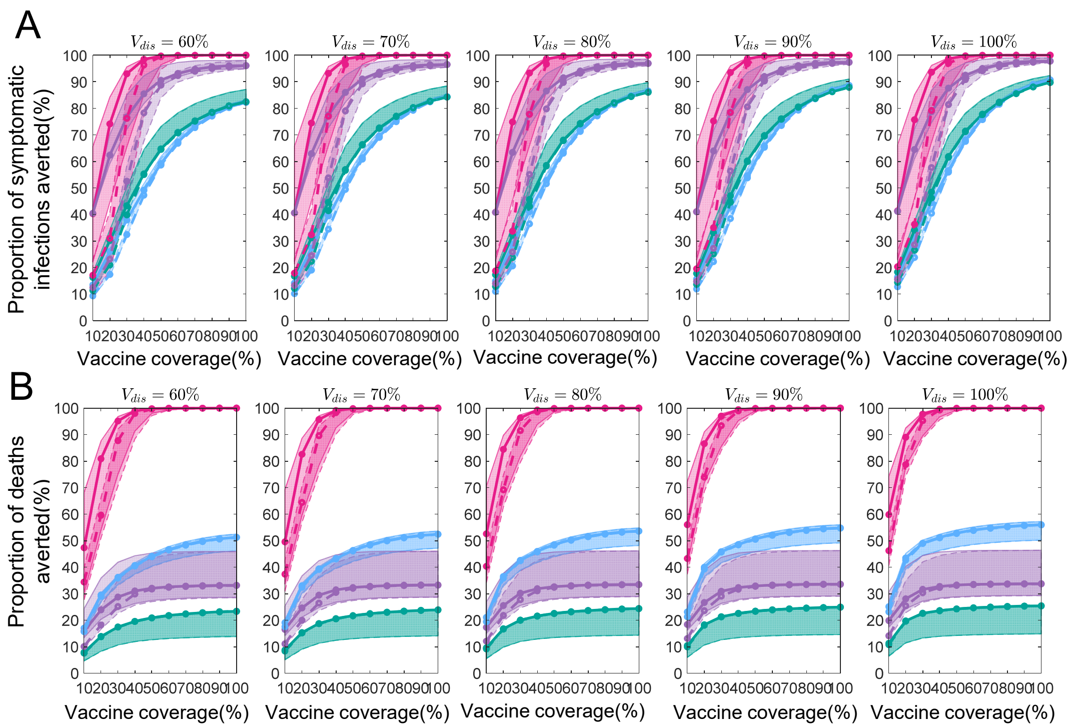

Figure S6: Percentage of symptomatic infections (A) and deaths (B) averted for both strategies under high-intensity non-pharmacological interventions. Solid lines with solid circles and dashed lines with hollow circles represent the near-optimal and pro-rata strategies, respectively. For clarity, we highlight the results of one simulation in each scenario. The shaded areas represent the results of multiple epidemiological landscape simulations. The shaded areas represent the range of effectiveness of strategies under multiple epidemiological landscapes simulated.

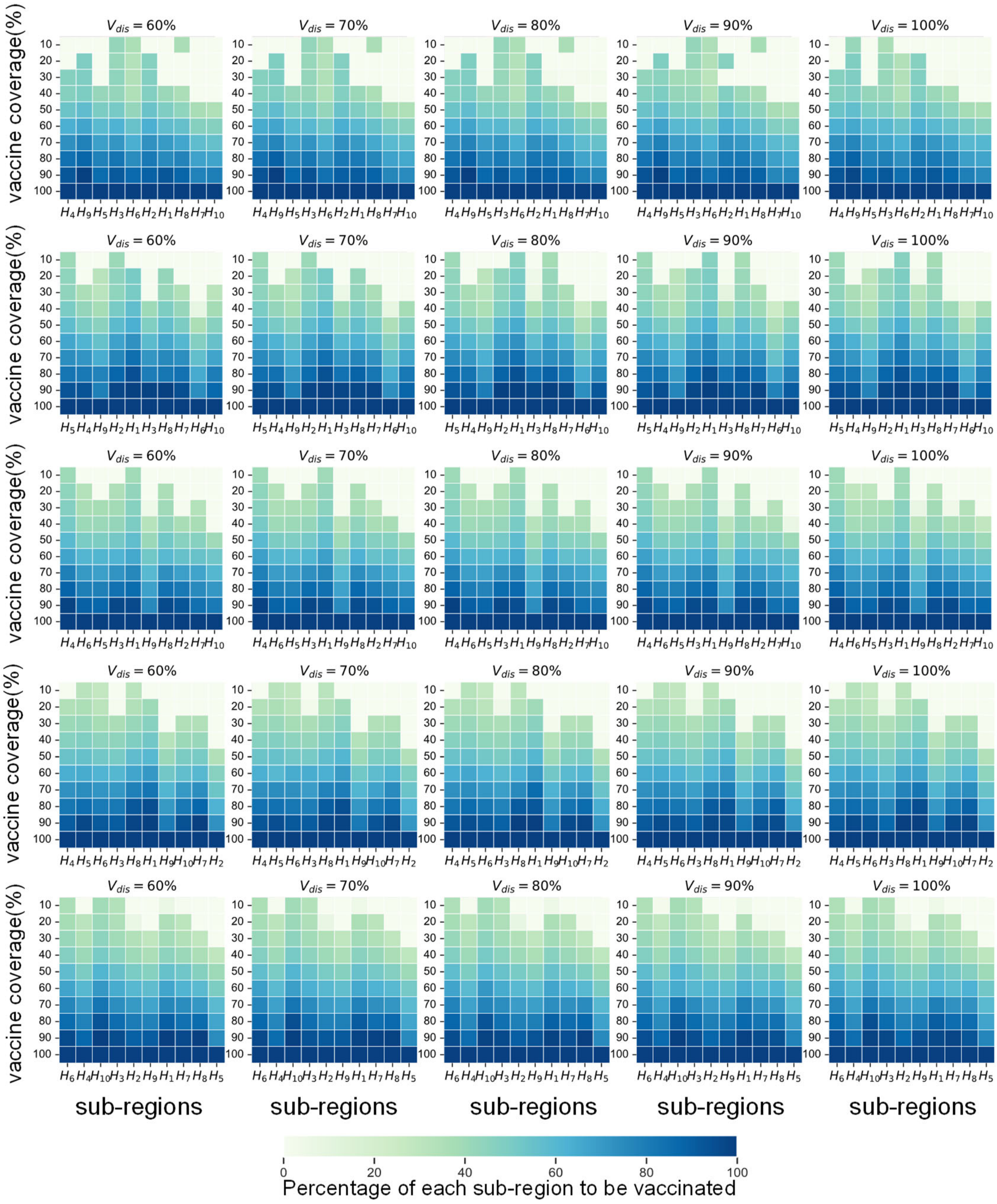

Figure S7: Near-optimal allocation strategies to minimize symptomatic infections under scenario 1 and high-intensity non-pharmacological interventions. For each heat map, each row from left to right is the decreasing direction of transmission risk, representing the total vaccine supply (percentage of the total population vaccinated) and each column represents a different subregion. Colors represent the percentage of the population in a sub-region to be vaccinated. For the whole figure, each row represents the result of one simulation.

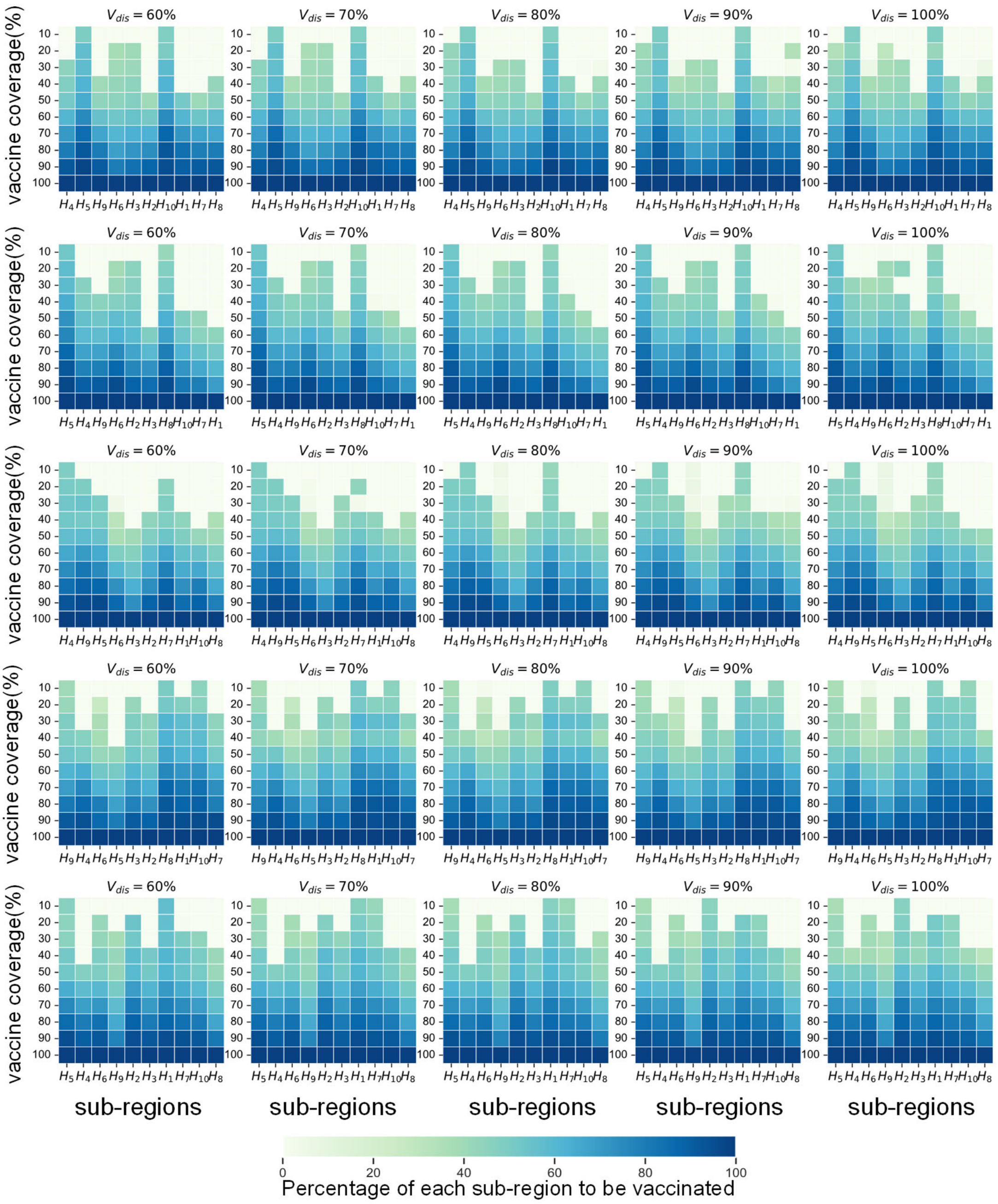

Figure S8: Near-optimal allocation strategies to minimize symptomatic infections under scenario 2 and high-intensity non-pharmacological interventions. For each heat map, each row from left to right is the decreasing direction of transmission risk, representing the total vaccine supply (percentage of the total population vaccinated) and each column represents a different subregion. Colors represent the percentage of the population in a sub-region to be vaccinated. For the whole figure, each row represents the result of one simulation.

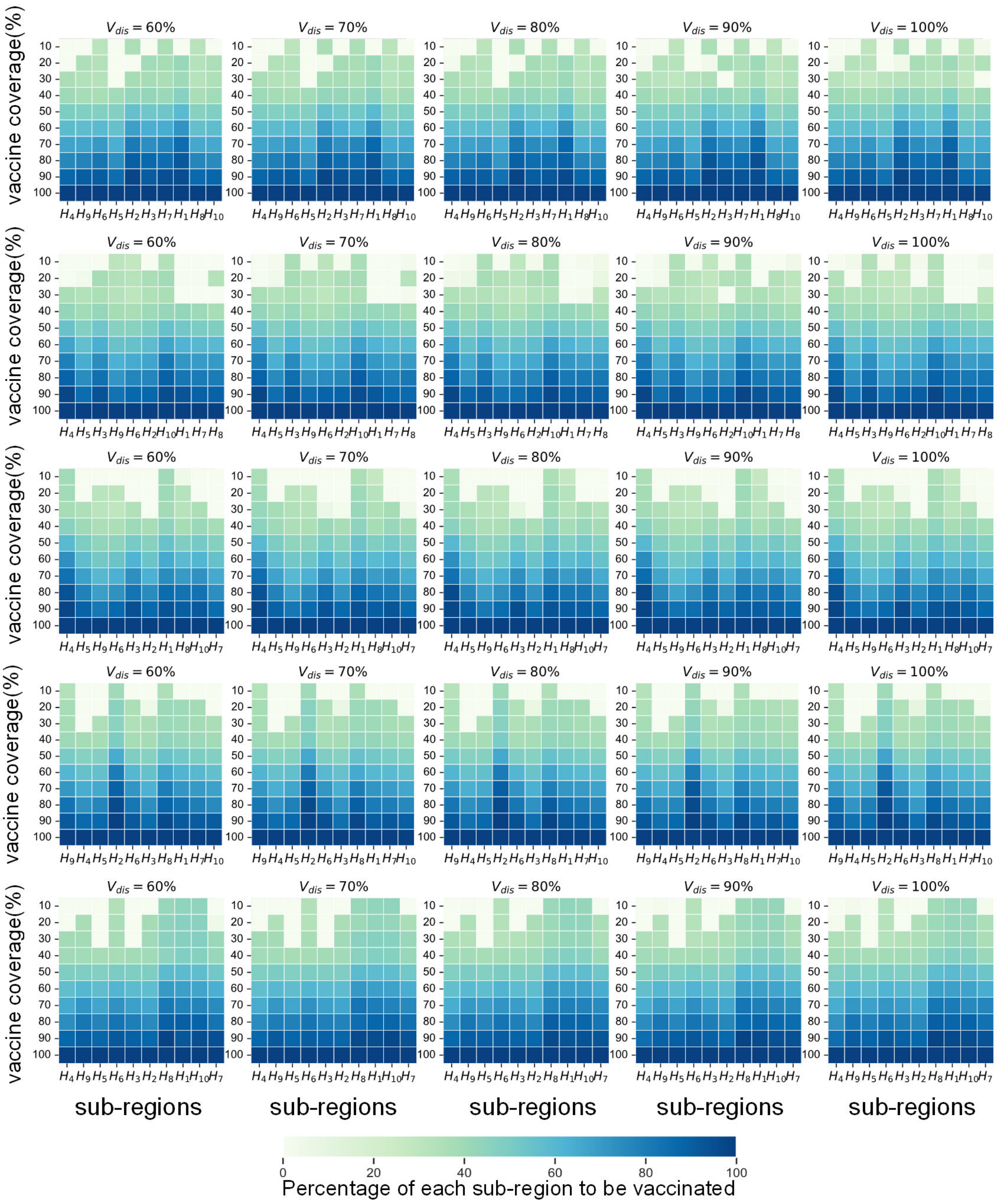

Figure S9: Near-optimal allocation strategies to minimize symptomatic infections under scenario 3 and high-intensity non-pharmacological interventions. For each heat map, each row from left to right is the decreasing direction of transmission risk, representing the total vaccine supply (percentage of the total population vaccinated) and each column represents a different subregion. Colors represent the percentage of the population in a sub-region to be vaccinated. For the whole figure, each row represents the result of one simulation.

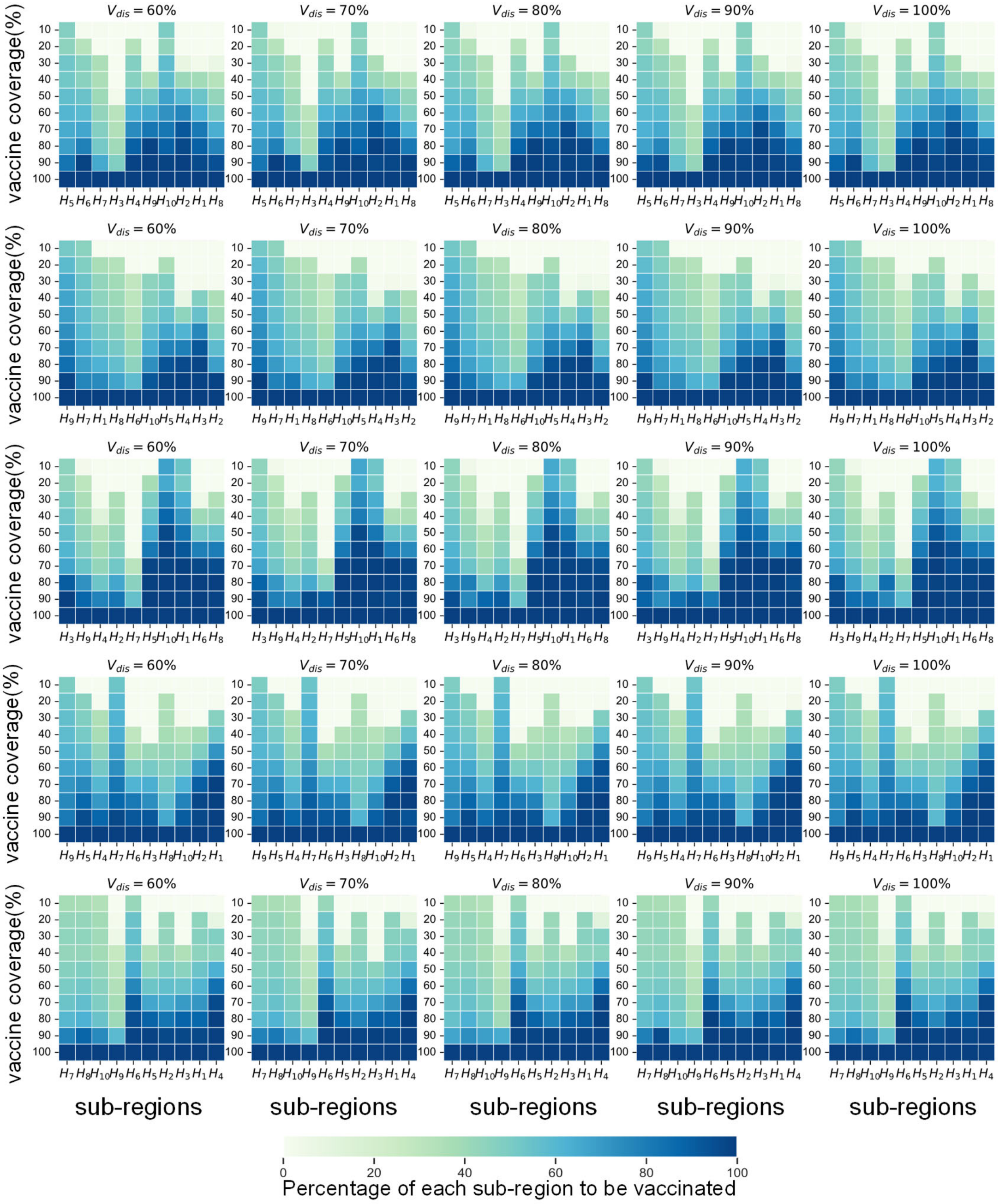

Figure S10: Near-optimal allocation strategies to minimize symptomatic infections under scenario 4 and high-intensity non-pharmacological interventions. For each heat map, each row from left to right is the decreasing direction of transmission risk, representing the total vaccine supply (percentage of the total population vaccinated) and each column represents a different subregion. Colors represent the percentage of the population in a sub-region to be vaccinated. For the whole figure, each row represents the result of one simulation.

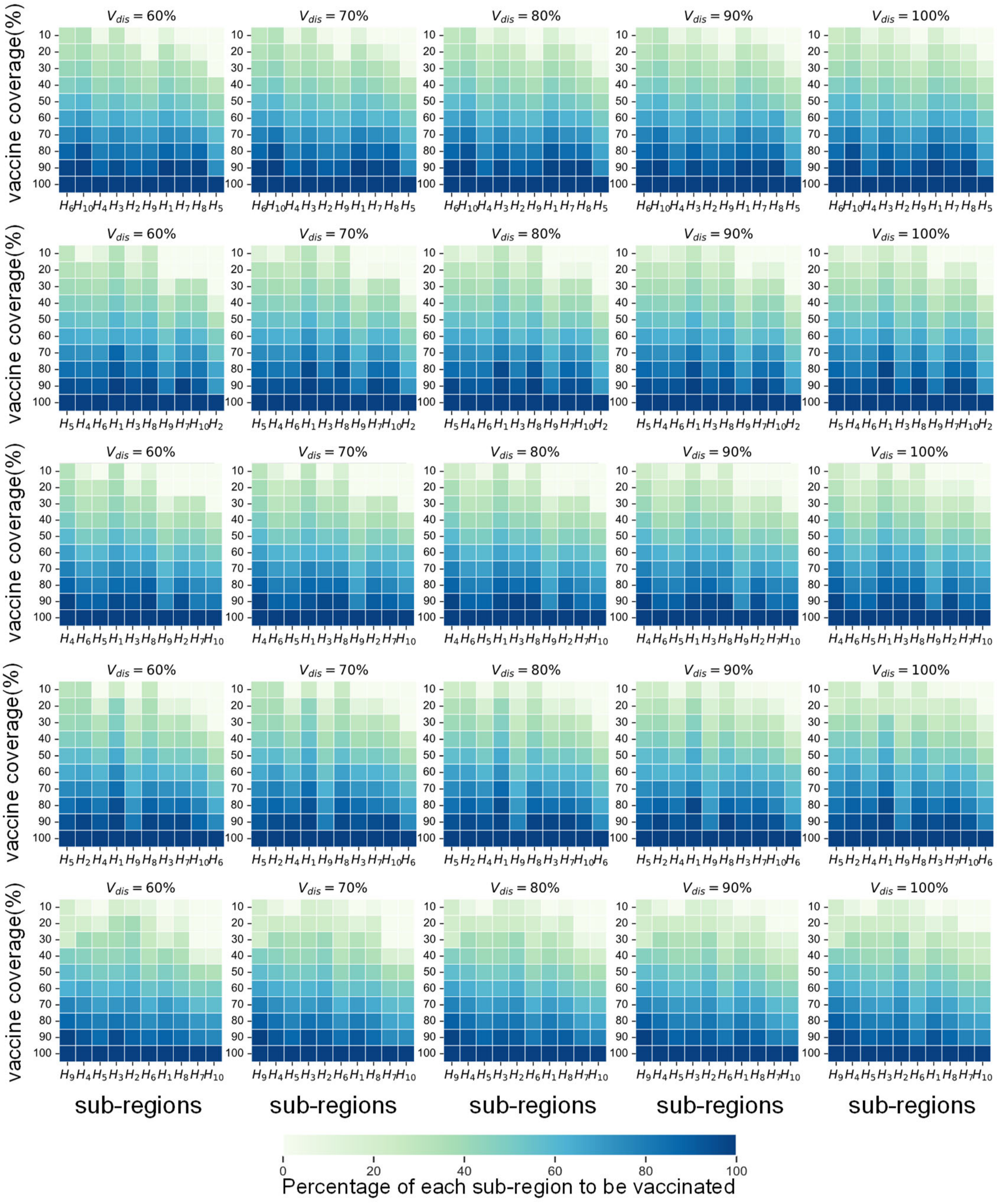

Figure S11: Near-optimal allocation strategies to minimize deaths under scenario 1 and high-intensity non-pharmacological interventions. For each heat map, each row from left to right is the decreasing direction of transmission risk, representing the total vaccine supply (percentage of the total population vaccinated) and each column represents a different subregion. Colors represent the percentage of the population in a sub-region to be vaccinated. For the whole figure, each row represents the result of one simulation.

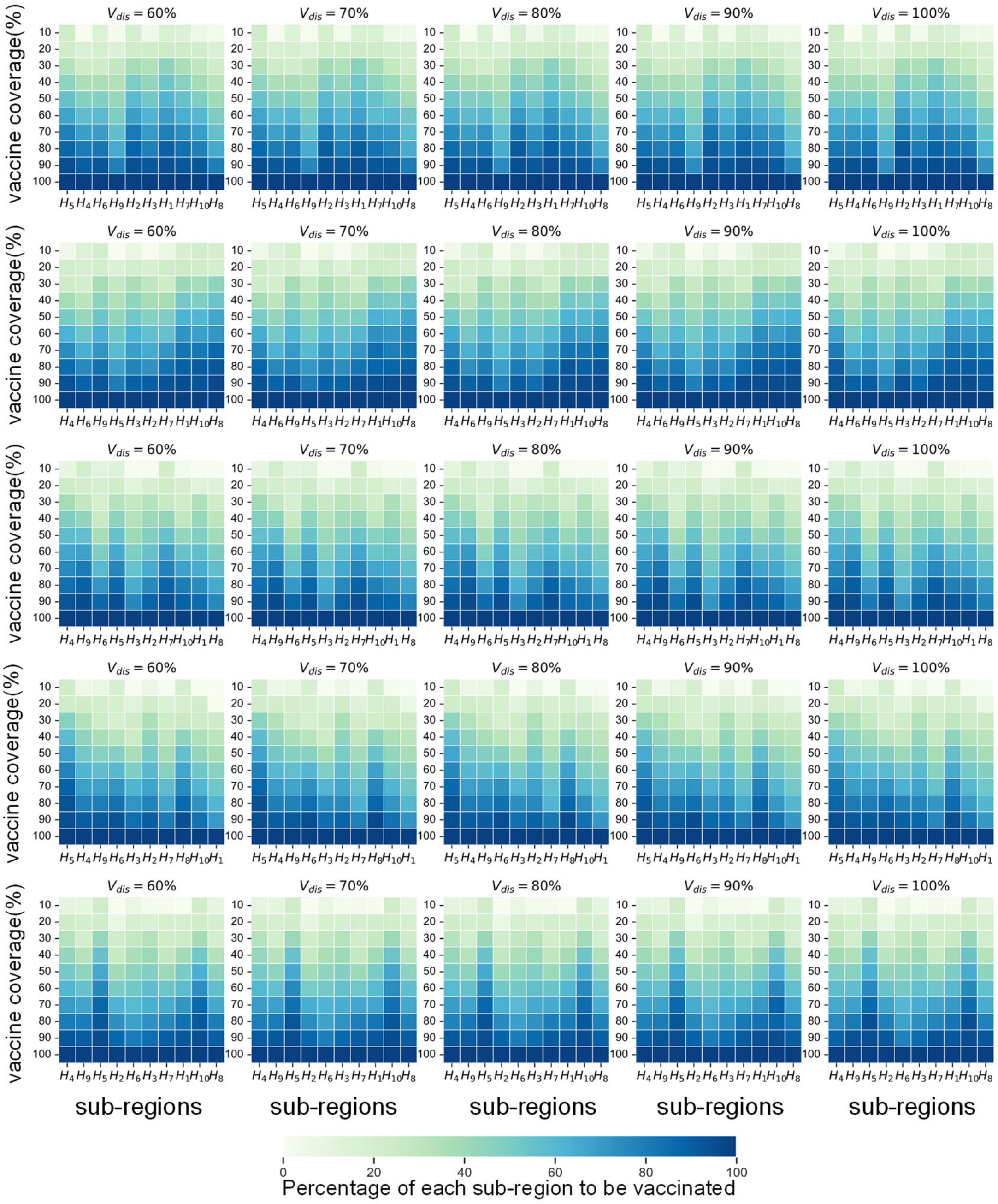

Figure S12: Near-optimal allocation strategies to minimize deaths under scenario 2 and high-intensity non-pharmacological interventions. For each heat map, each row from left to right is the decreasing direction of transmission risk, representing the total vaccine supply (percentage of the total population vaccinated) and each column represents a different subregion. Colors represent the percentage of the population in a sub-region to be vaccinated. For the whole figure, each row represents the result of one simulation.

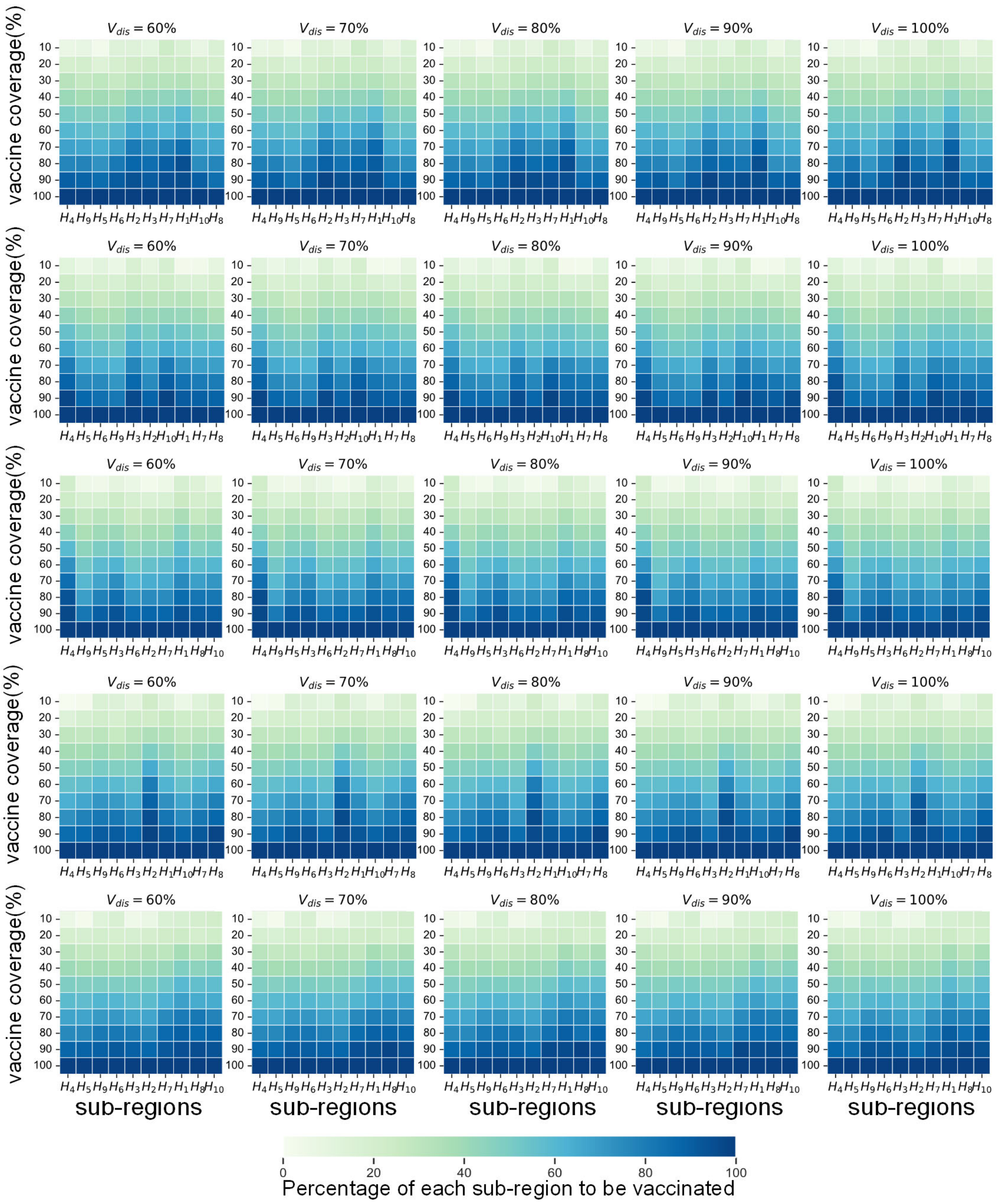

Figure S13: Near-optimal allocation strategies to minimize deaths under scenario 3 and high-intensity non-pharmacological interventions. For each heat map, each row from left to right is the decreasing direction of transmission risk, representing the total vaccine supply (percentage of the total population vaccinated) and each column represents a different subregion. Colors represent the percentage of the population in a sub-region to be vaccinated. For the whole figure, each row represents the result of one simulation.

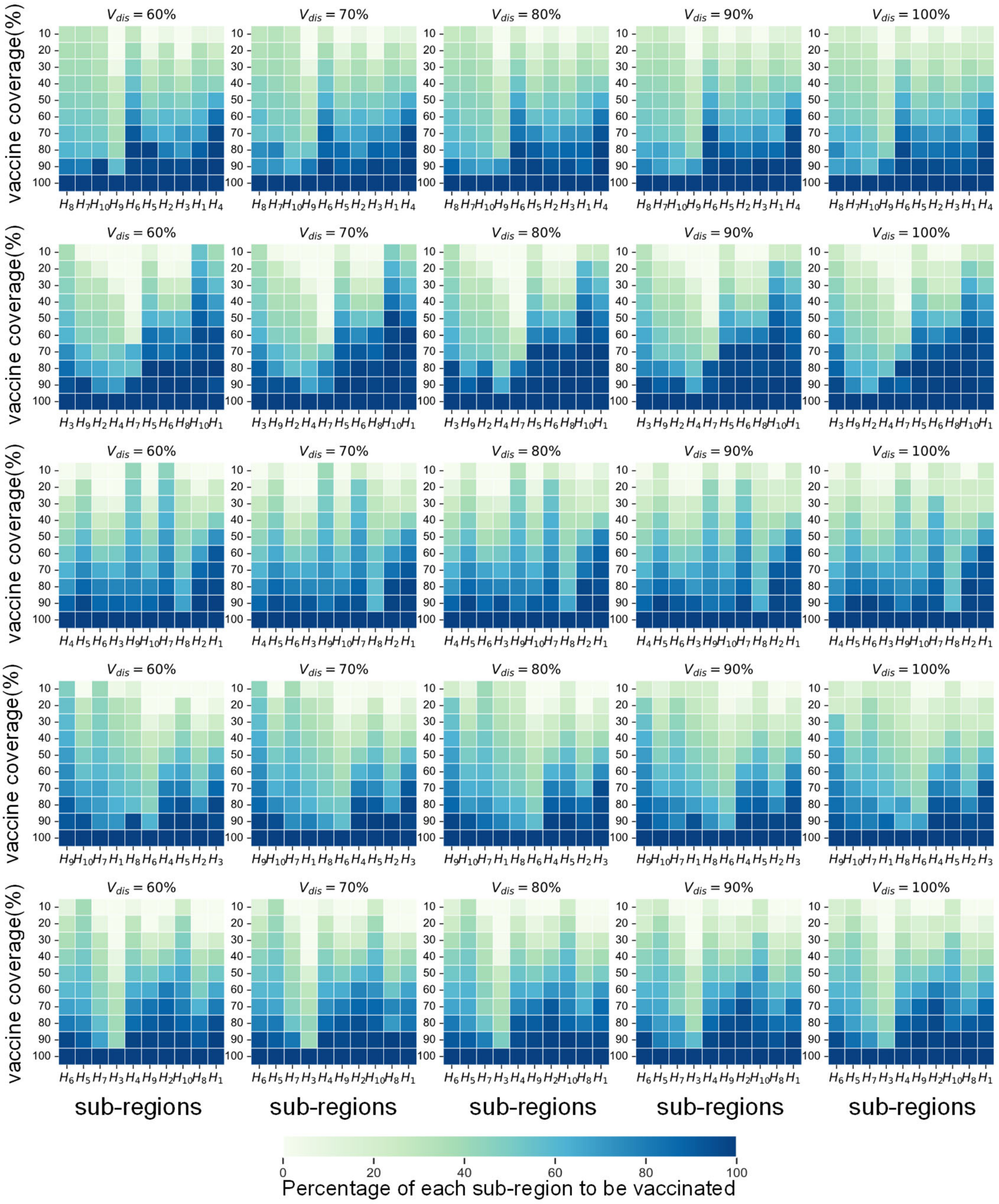

Figure S14: Near-optimal allocation strategies to minimize deaths under scenario 4 and high-intensity non-pharmacological interventions. For each heat map, each row from left to right is the decreasing direction of transmission risk, representing the total vaccine supply (percentage of the total population vaccinated) and each column represents a different subregion. Colors represent the percentage of the population in a sub-region to be vaccinated. For the whole figure, each row represents the result of one simulation.

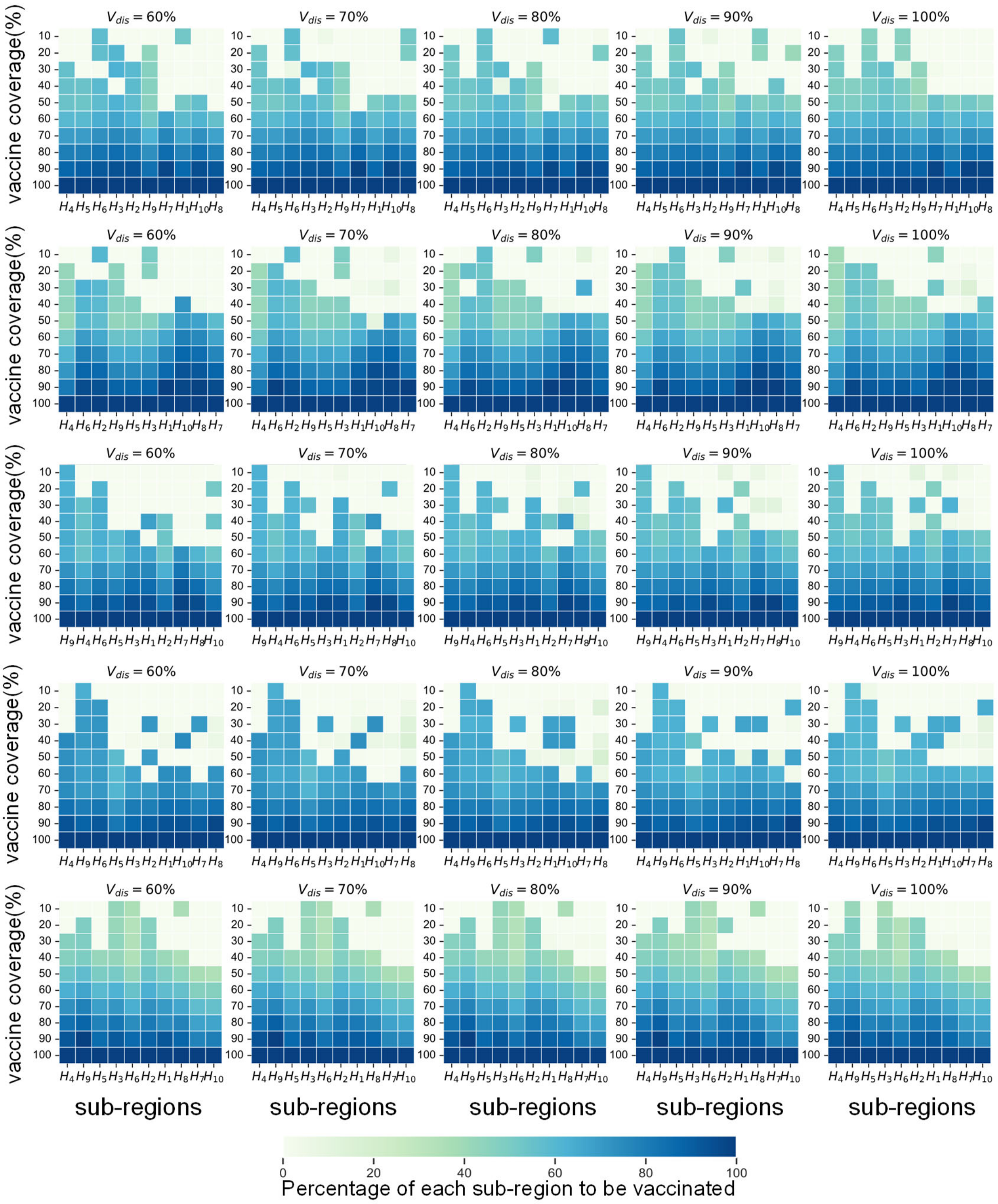

Figure S15: Near-optimal allocation strategies to minimize symptomatic infections under scenario 1 and moderate-intensity non-pharmacological interventions. For each heat map, each row from left to right is the decreasing direction of transmission risk, representing the total vaccine supply (percentage of the total population vaccinated) and each column represents a different subregion. Colors represent the percentage of the population in a sub-region to be vaccinated. For the whole figure, each row represents the result of one simulation.

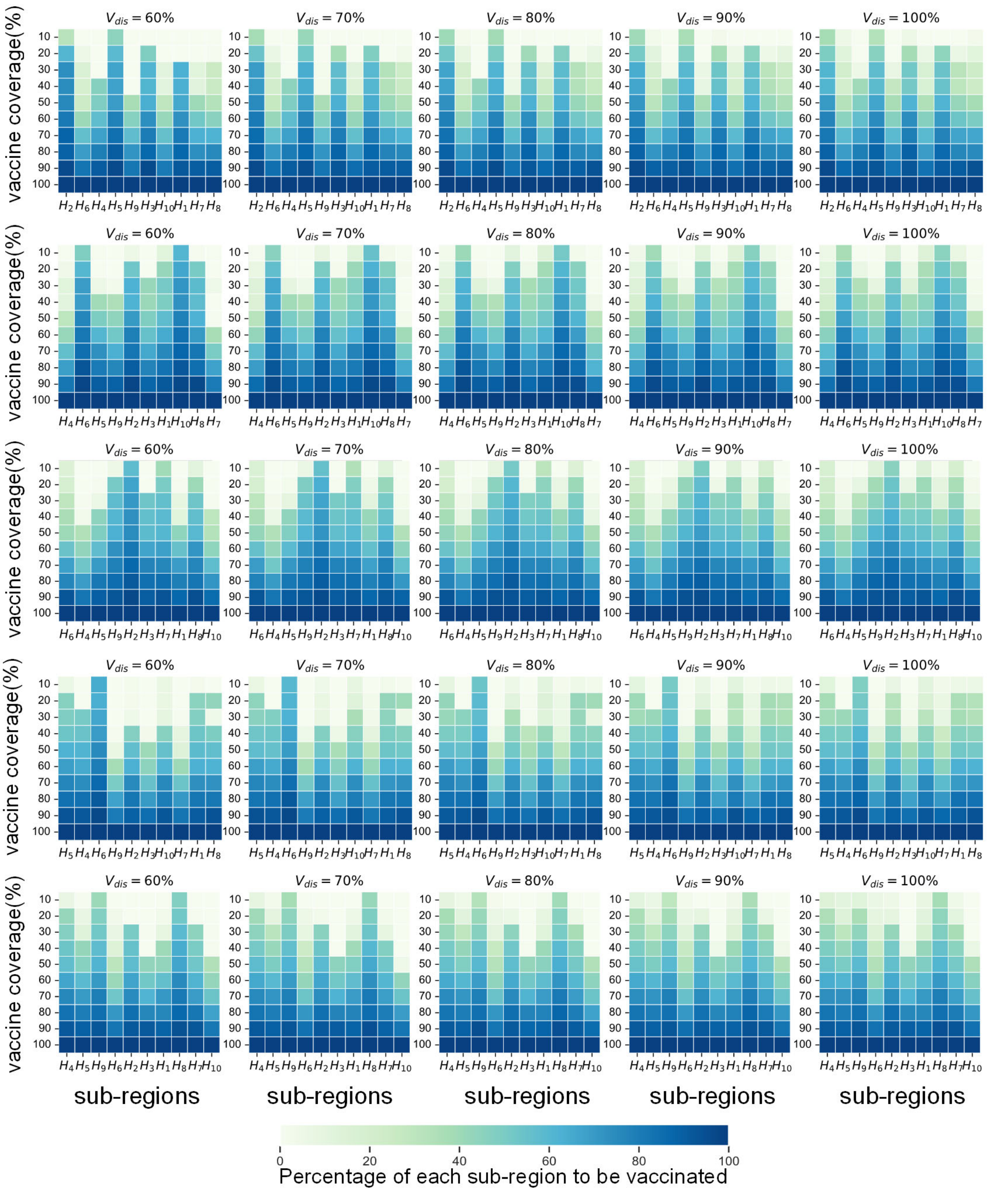

Figure S16: Near-optimal allocation strategies to minimize symptomatic infections under scenario 2 and moderate-intensity non-pharmacological interventions. For each heat map, each row from left to right is the decreasing direction of transmission risk, representing the total vaccine supply (percentage of the total population vaccinated) and each column represents a different subregion. Colors represent the percentage of the population in a sub-region to be vaccinated. For the whole figure, each row represents the result of one simulation.

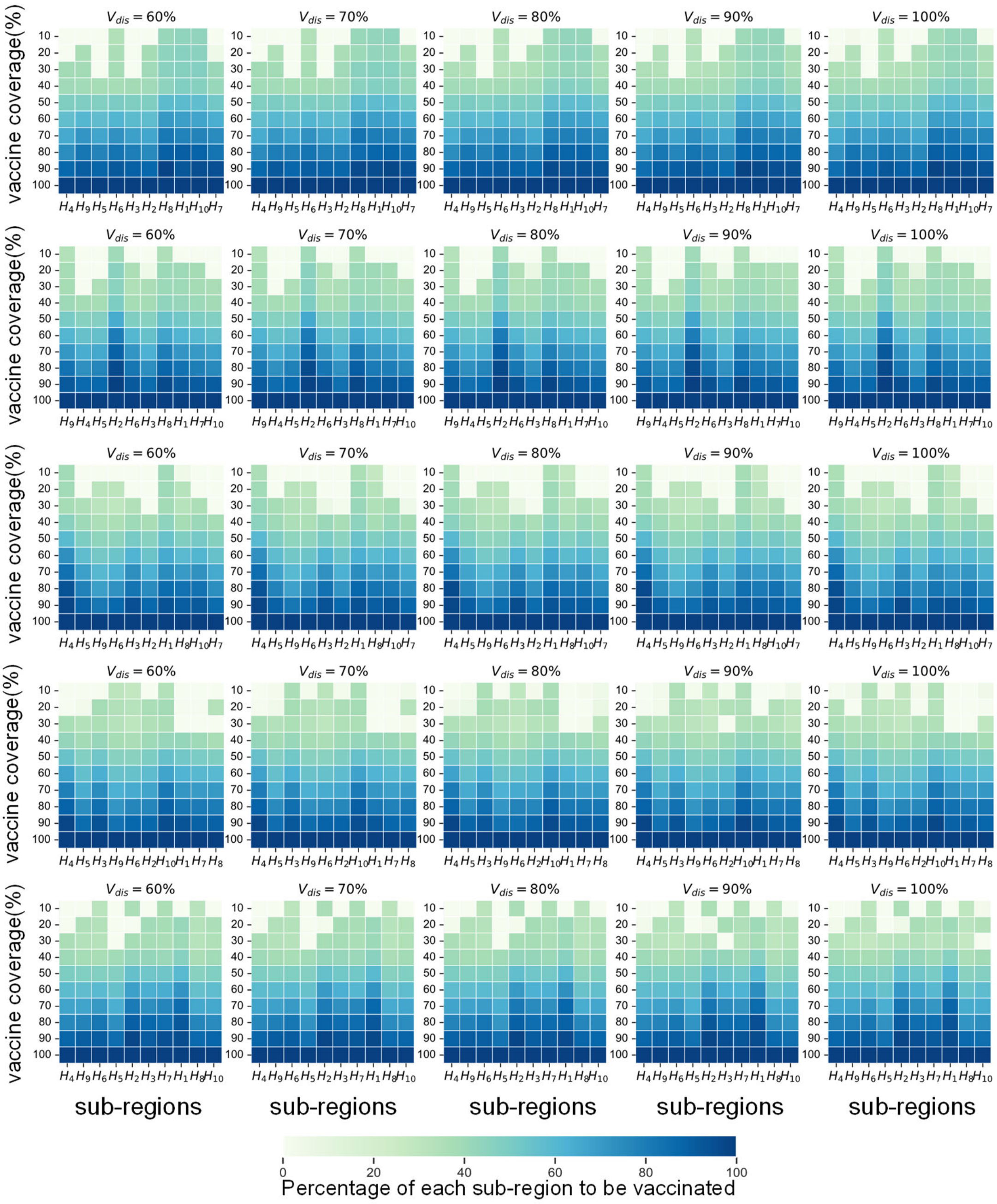

Figure S17: Near-optimal allocation strategies to minimize symptomatic infections under scenario 3 and moderate-intensity non-pharmacological interventions. For each heat map, each row from left to right is the decreasing direction of transmission risk, representing the total vaccine supply (percentage of the total population vaccinated) and each column represents a different subregion. Colors represent the percentage of the population in a sub-region to be vaccinated. For the whole figure, each row represents the result of one simulation.

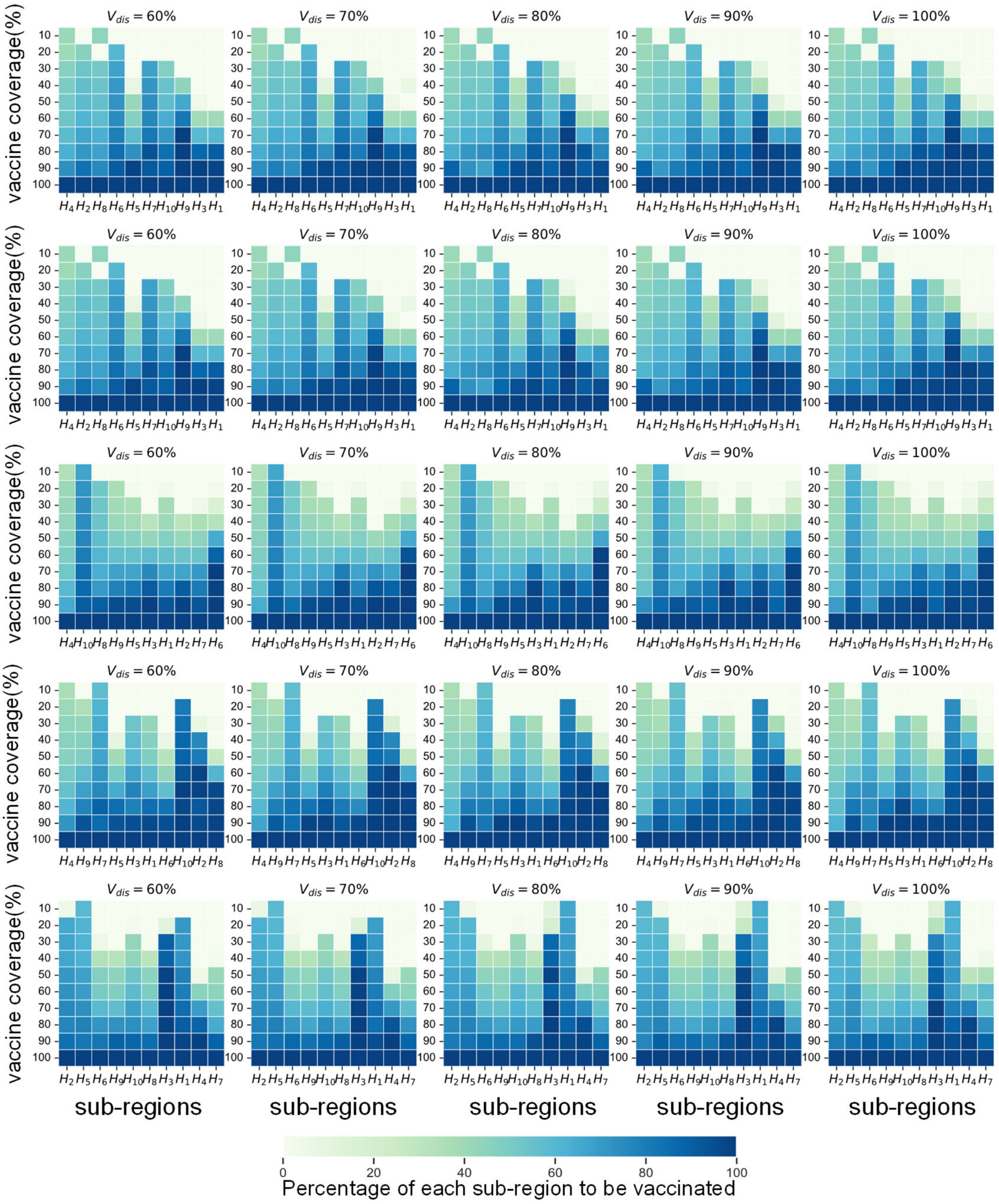

Figure S18: Near-optimal allocation strategies to minimize symptomatic infections under scenario 4 and moderate-intensity non-pharmacological interventions. For each heat map, each row from left to right is the decreasing direction of transmission risk, representing the total vaccine supply (percentage of the total population vaccinated) and each column represents a different subregion. Colors represent the percentage of the population in a sub-region to be vaccinated. For the whole figure, each row represents the result of one simulation.

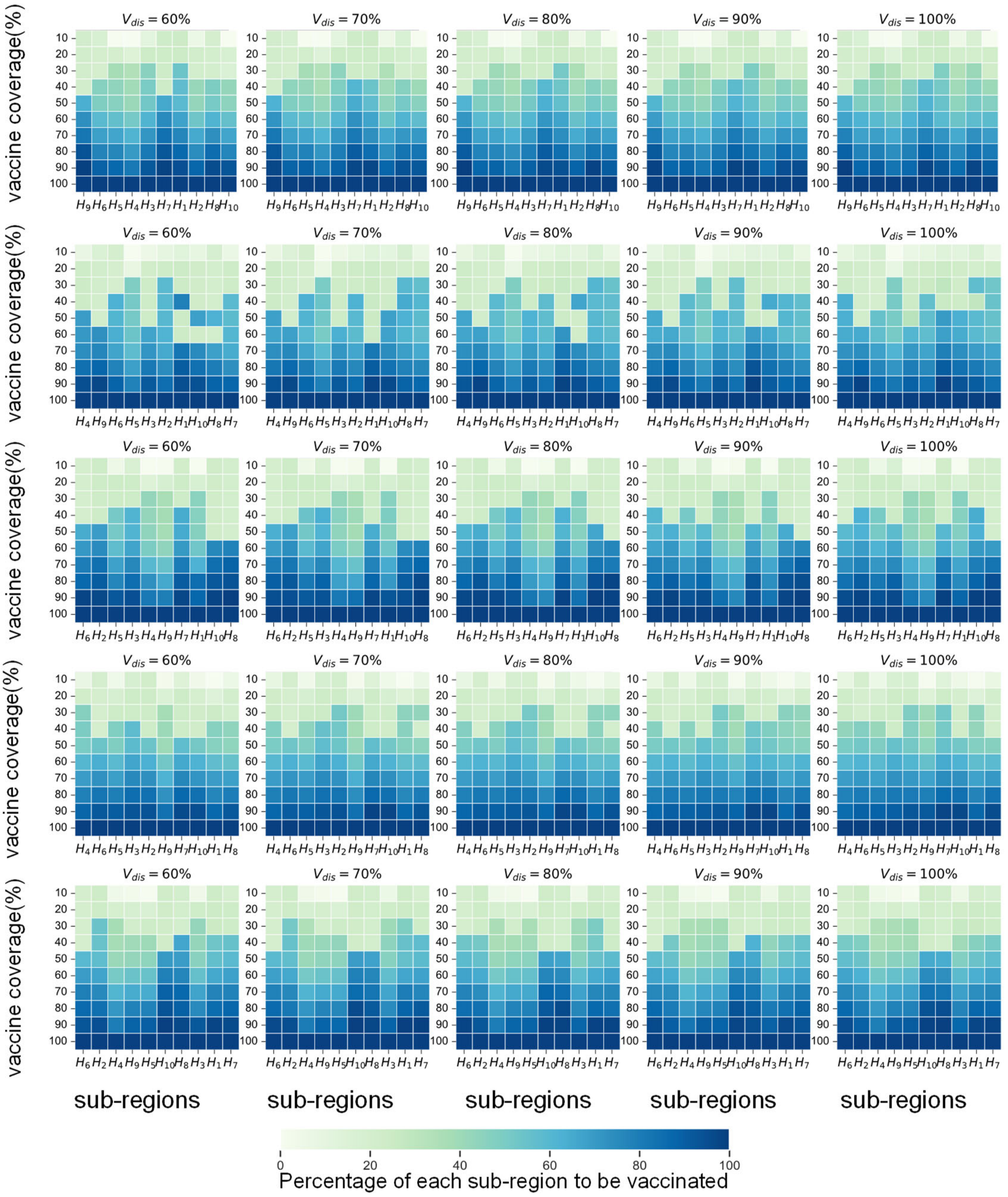

Figure S19: Near-optimal allocation strategies to minimize deaths under scenario 1 and moderate-intensity non-pharmacological interventions. For each heat map, each row from left to right is the decreasing direction of transmission risk, representing the total vaccine supply (percentage of the total population vaccinated) and each column represents a different subregion. Colors represent the percentage of the population in a sub-region to be vaccinated. For the whole figure, each row represents the result of one simulation.

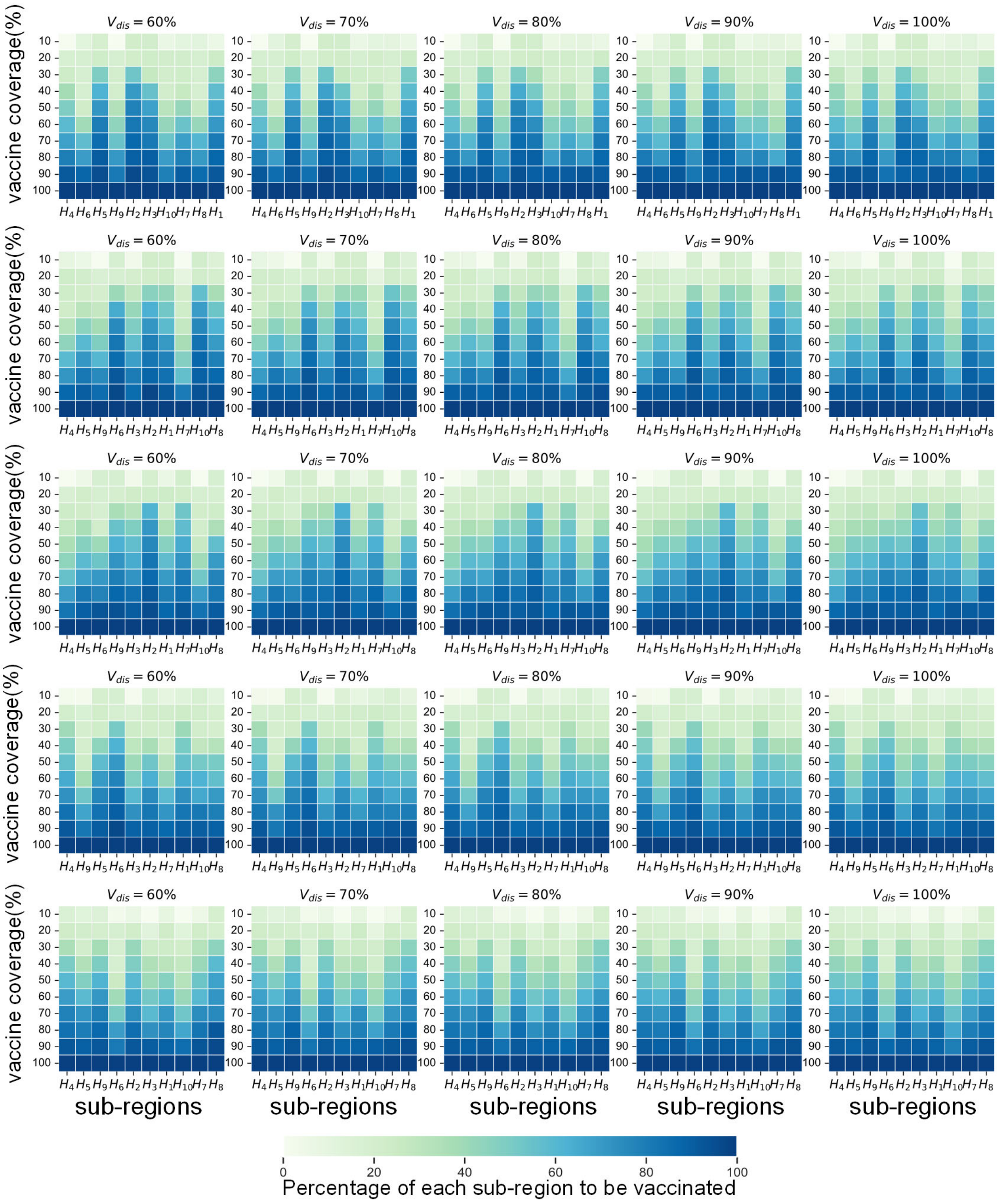

Figure S20: Near-optimal allocation strategies to minimize deaths under scenario 2 and moderate-intensity non-pharmacological interventions. For each heat map, each row from left to right is the decreasing direction of transmission risk, representing the total vaccine supply (percentage of the total population vaccinated) and each column represents a different subregion. Colors represent the percentage of the population in a sub-region to be vaccinated. For the whole figure, each row represents the result of one simulation.

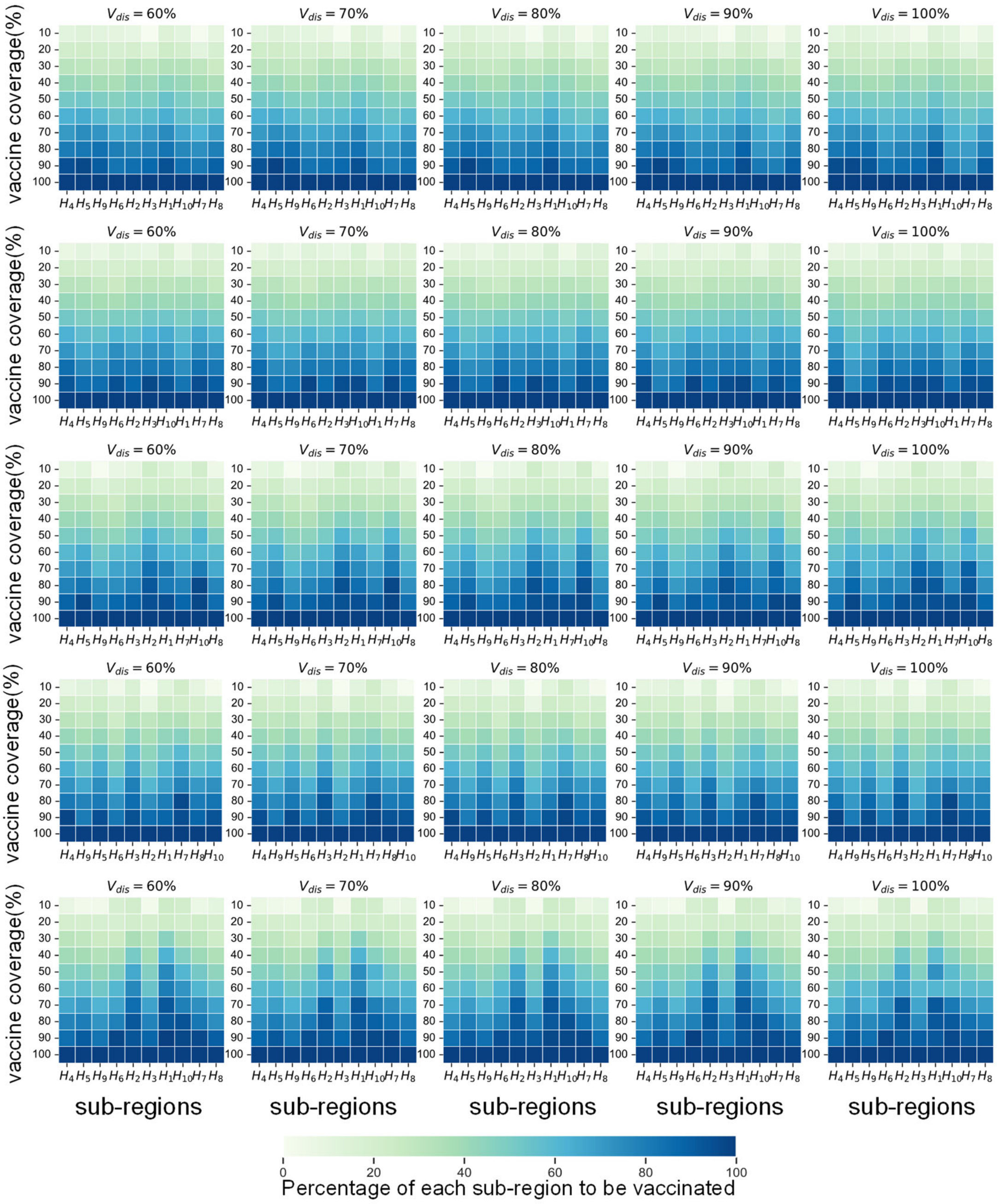

Figure S21: Near-optimal allocation strategies to minimize deaths under scenario 3 and moderate-intensity non-pharmacological interventions. For each heat map, each row from left to right is the decreasing direction of transmission risk, representing the total vaccine supply (percentage of the total population vaccinated) and each column represents a different subregion. Colors represent the percentage of the population in a sub-region to be vaccinated. For the whole figure, each row represents the result of one simulation.

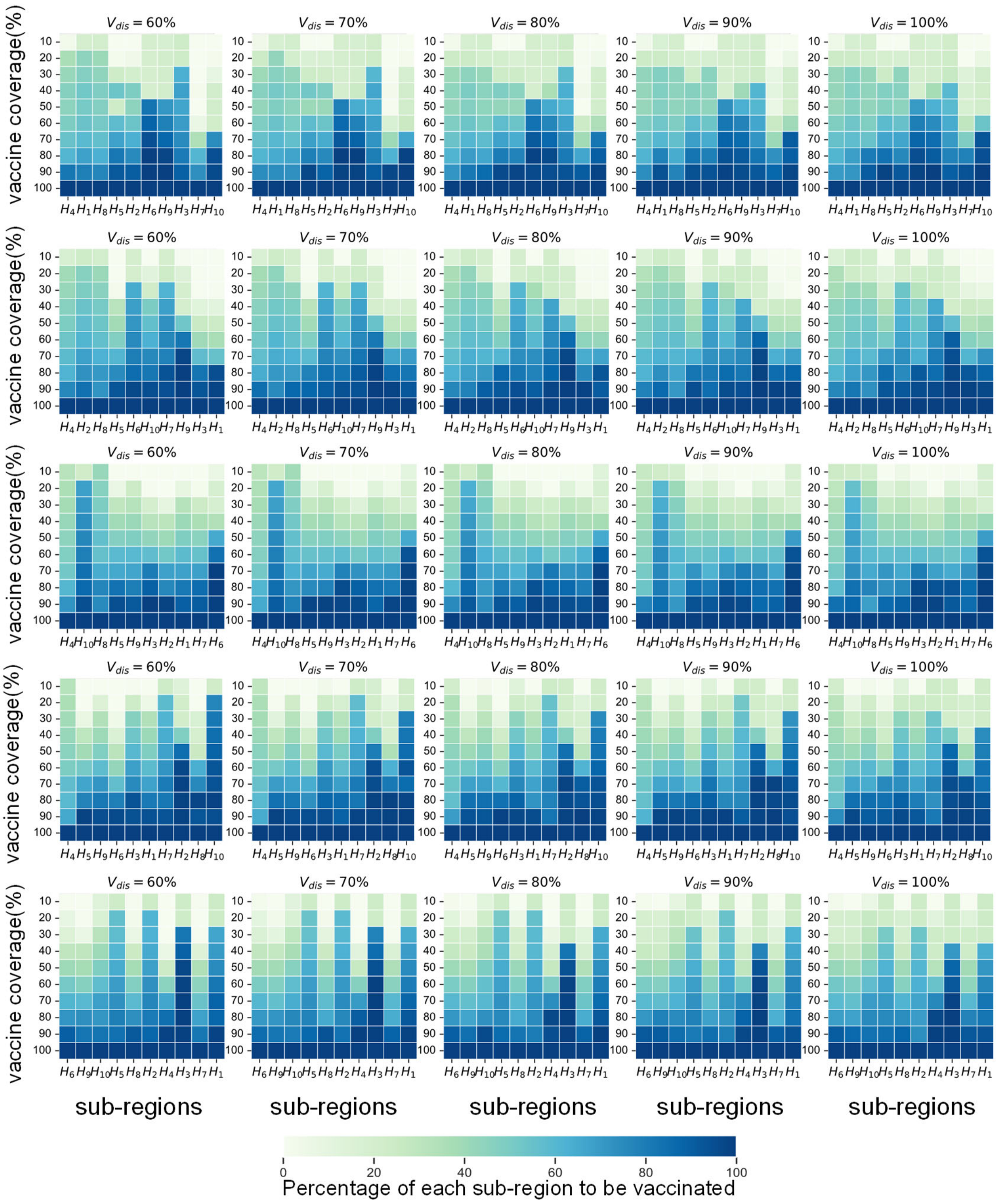

Figure S22: Near-optimal allocation strategies to minimize deaths under scenario 4 and moderate-intensity non-pharmacological interventions. For each heat map, each row from left to right is the decreasing direction of transmission risk, representing the total vaccine supply (percentage of the total population vaccinated) and each column represents a different subregion. Colors represent the percentage of the population in a sub-region to be vaccinated. For the whole figure, each row represents the result of one simulation.

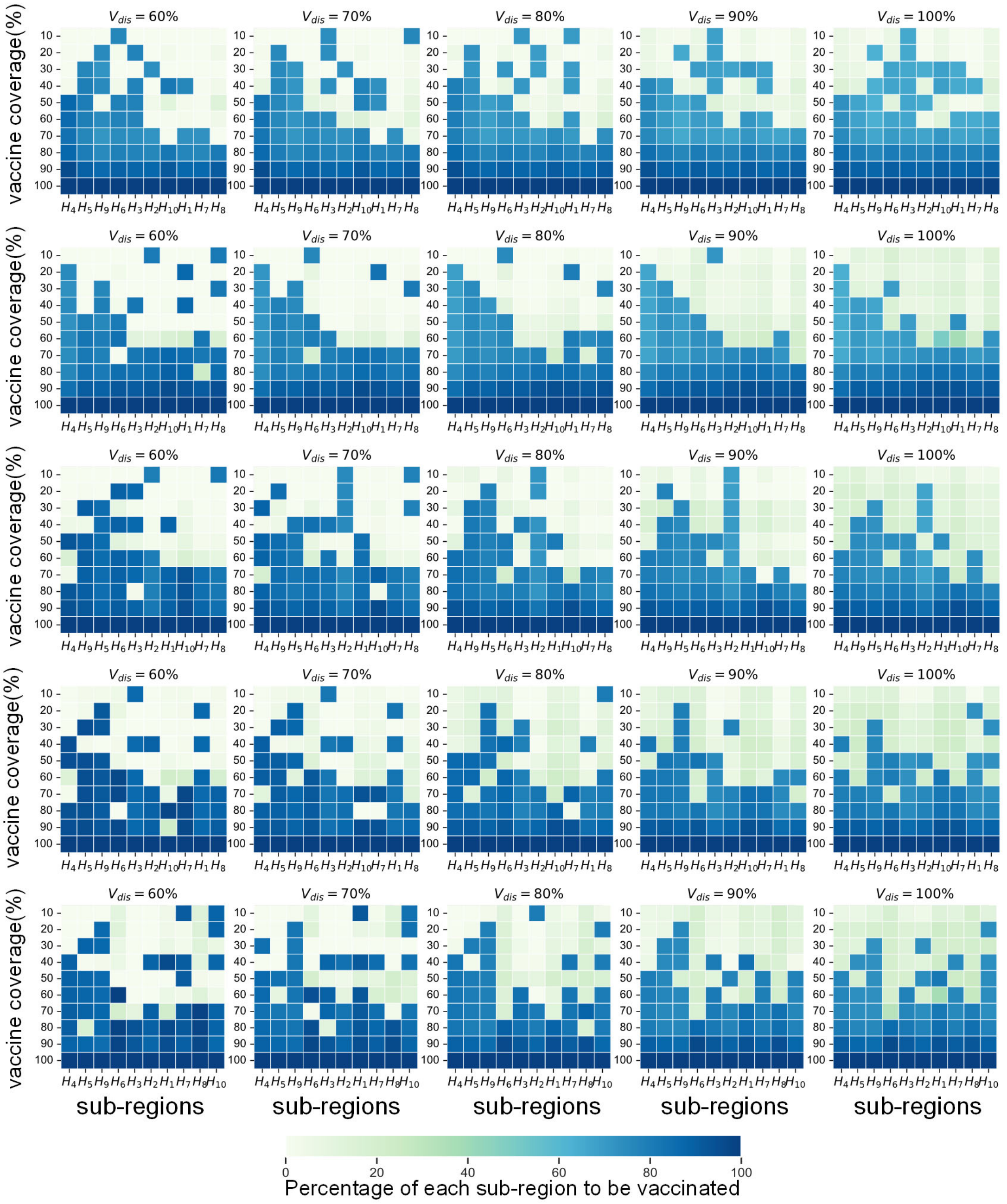

Figure S23: Near-optimal allocation strategies to minimize symptomatic infections under scenario 1 and low-intensity non-pharmacological interventions. For each heat map, each row from left to right is the decreasing direction of transmission risk, representing the total vaccine supply (percentage of the total population vaccinated) and each column represents a different subregion. Colors represent the percentage of the population in a sub-region to be vaccinated. For the whole figure, each row represents the result of one simulation.

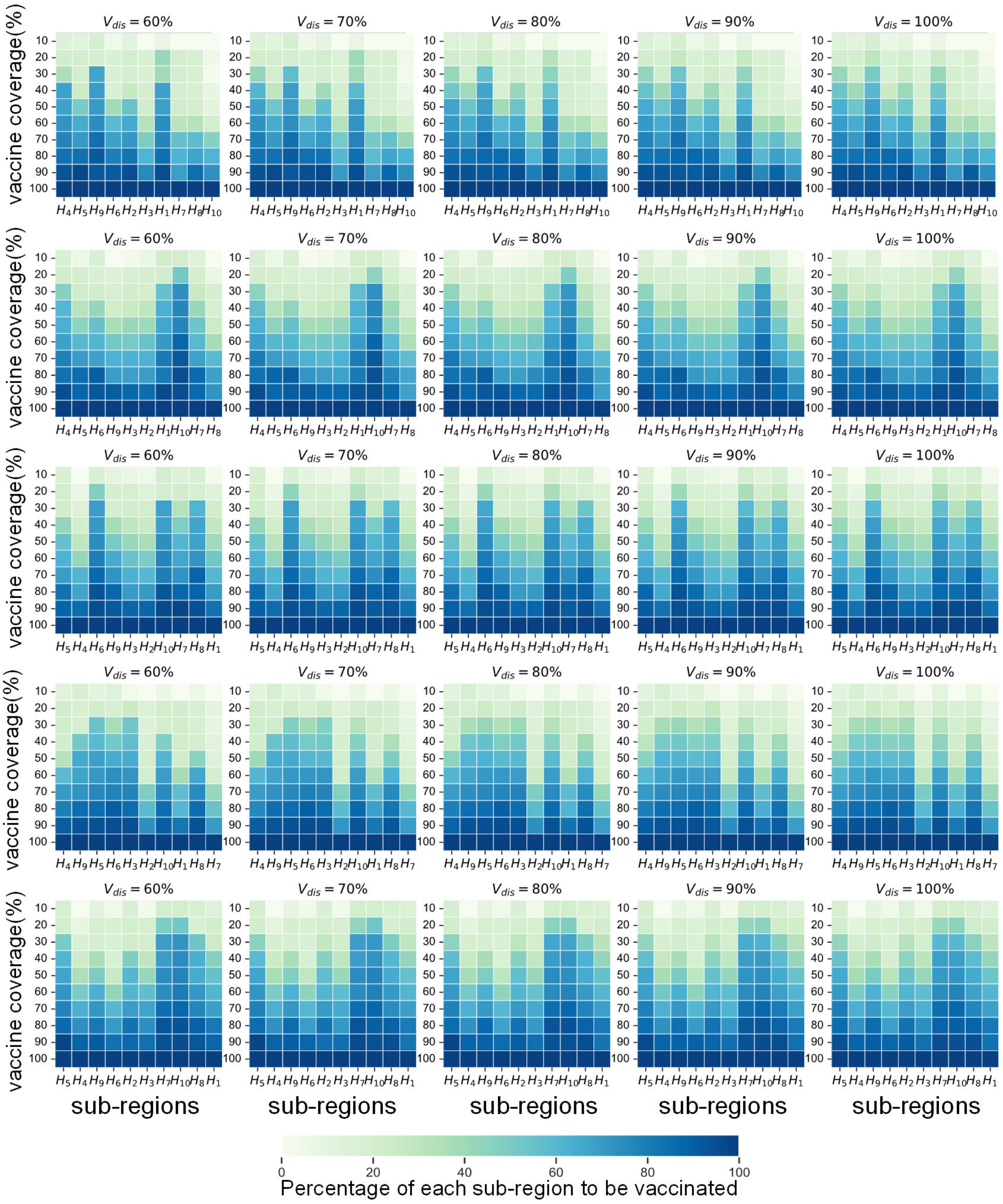

Figure S24: Near-optimal allocation strategies to minimize symptomatic infections under scenario 2 and low-intensity non-pharmacological interventions. For each heat map, each row from left to right is the decreasing direction of transmission risk, representing the total vaccine supply (percentage of the total population vaccinated) and each column represents a different subregion. Colors represent the percentage of the population in a sub-region to be vaccinated. For the whole figure, each row represents the result of one simulation.

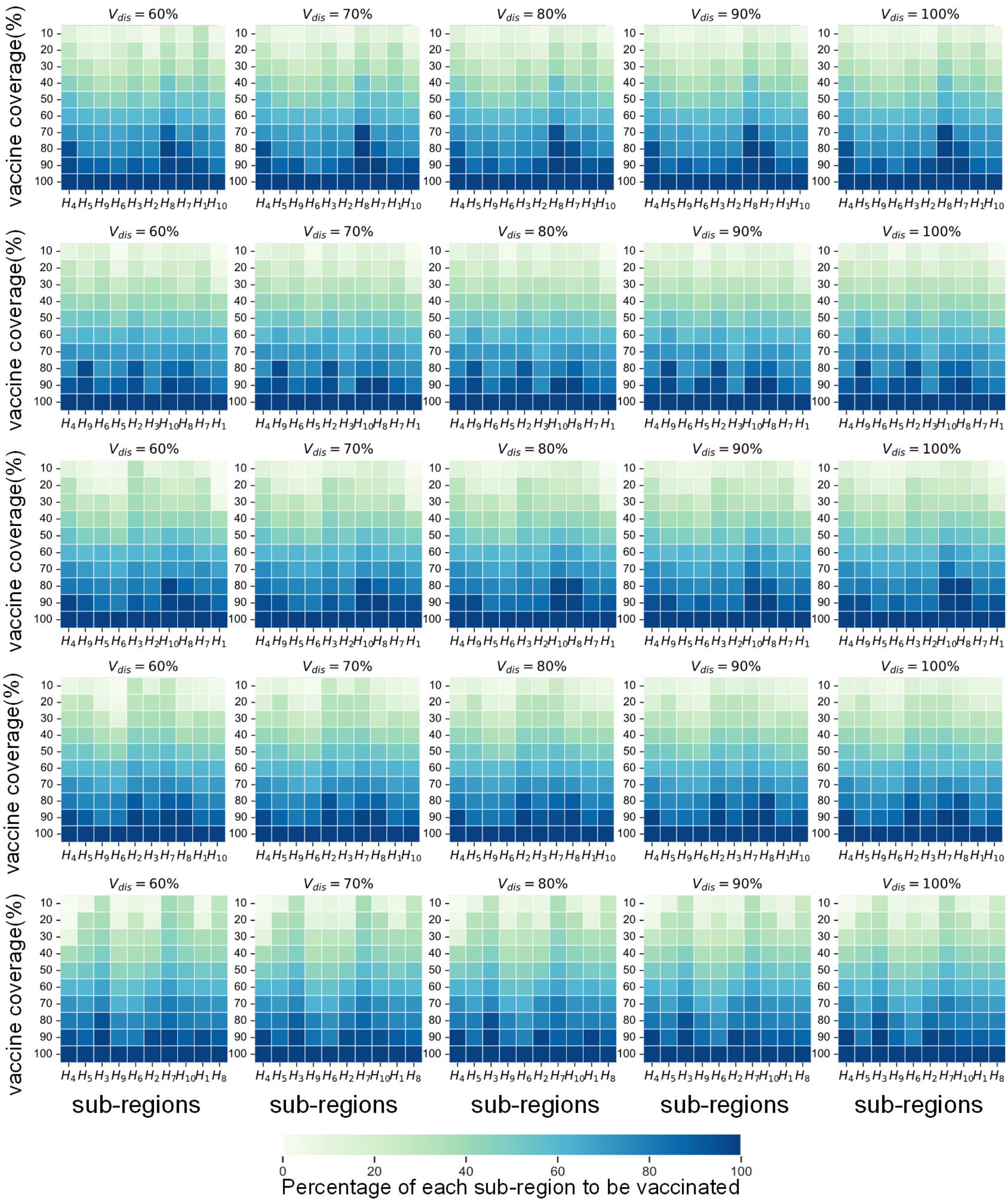

Figure S25: Near-optimal allocation strategies to minimize symptomatic infections under scenario 3 and low-intensity non-pharmacological interventions. For each heat map, each row from left to right is the decreasing direction of transmission risk, representing the total vaccine supply (percentage of the total population vaccinated) and each column represents a different subregion. Colors represent the percentage of the population in a sub-region to be vaccinated. For the whole figure, each row represents the result of one simulation.

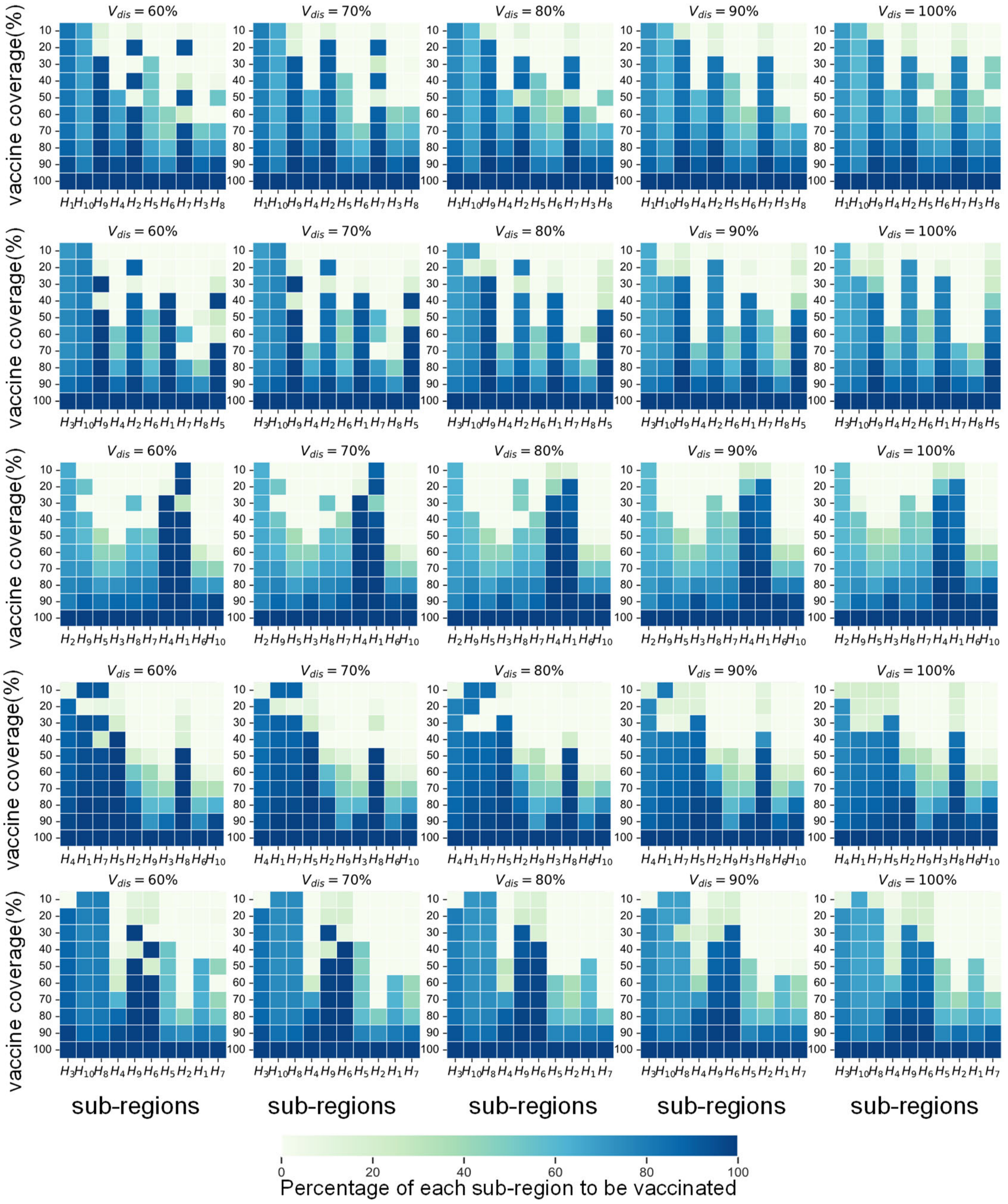

Figure S26: Near-optimal allocation strategies to minimize symptomatic infections under scenario 4 and low-intensity non-pharmacological interventions. For each heat map, each row from left to right is the decreasing direction of transmission risk, representing the total vaccine supply (percentage of the total population vaccinated) and each column represents a different subregion. Colors represent the percentage of the population in a sub-region to be vaccinated. For the whole figure, each row represents the result of one simulation.

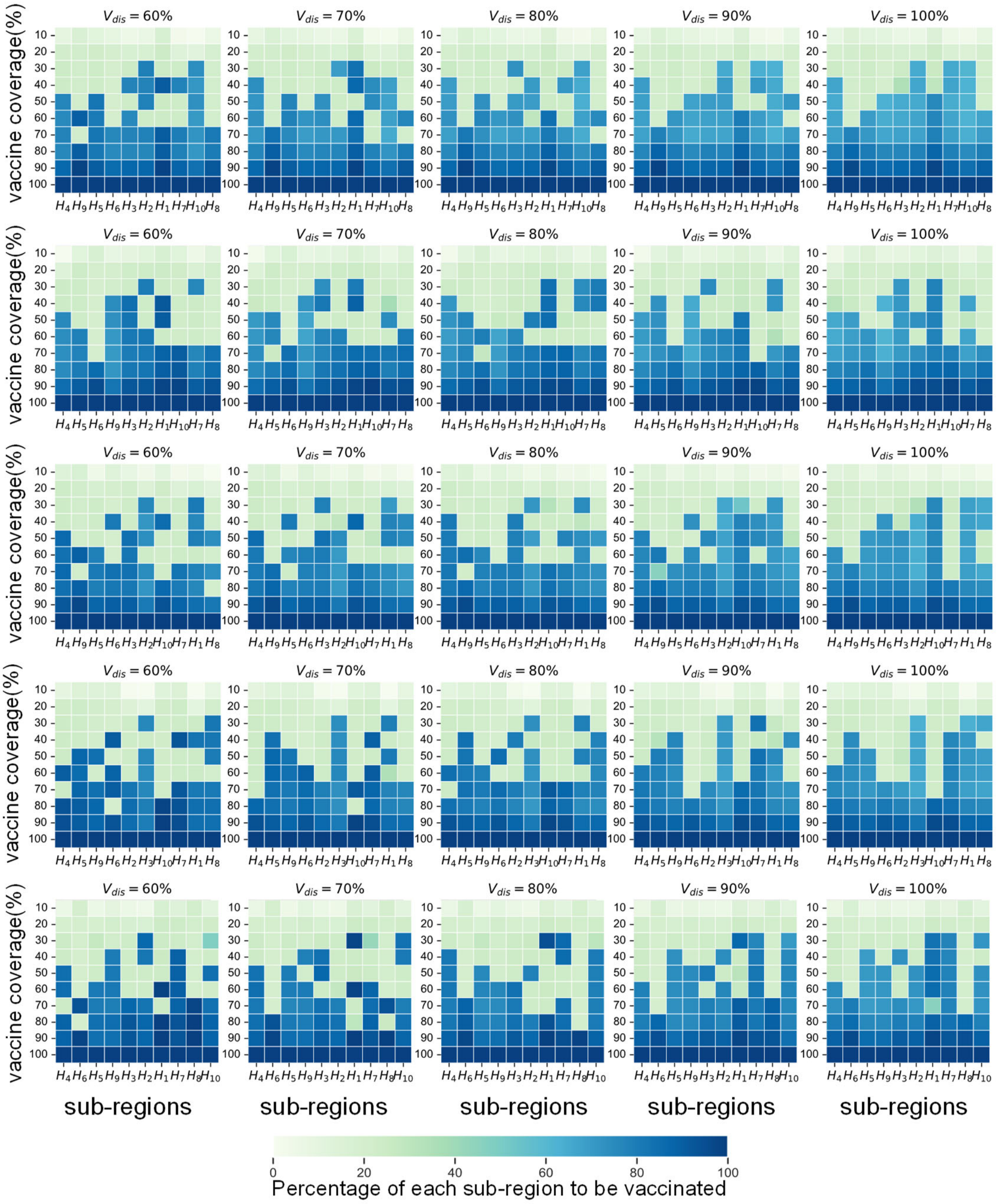

Figure S27: Near-optimal allocation strategies to minimize deaths under scenario 1 and low-intensity non-pharmacological interventions. For each heat map, each row from left to right is the decreasing direction of transmission risk, representing the total vaccine supply (percentage of the total population vaccinated) and each column represents a different subregion. Colors represent the percentage of the population in a sub-region to be vaccinated. For the whole figure, each row represents the result of one simulation.

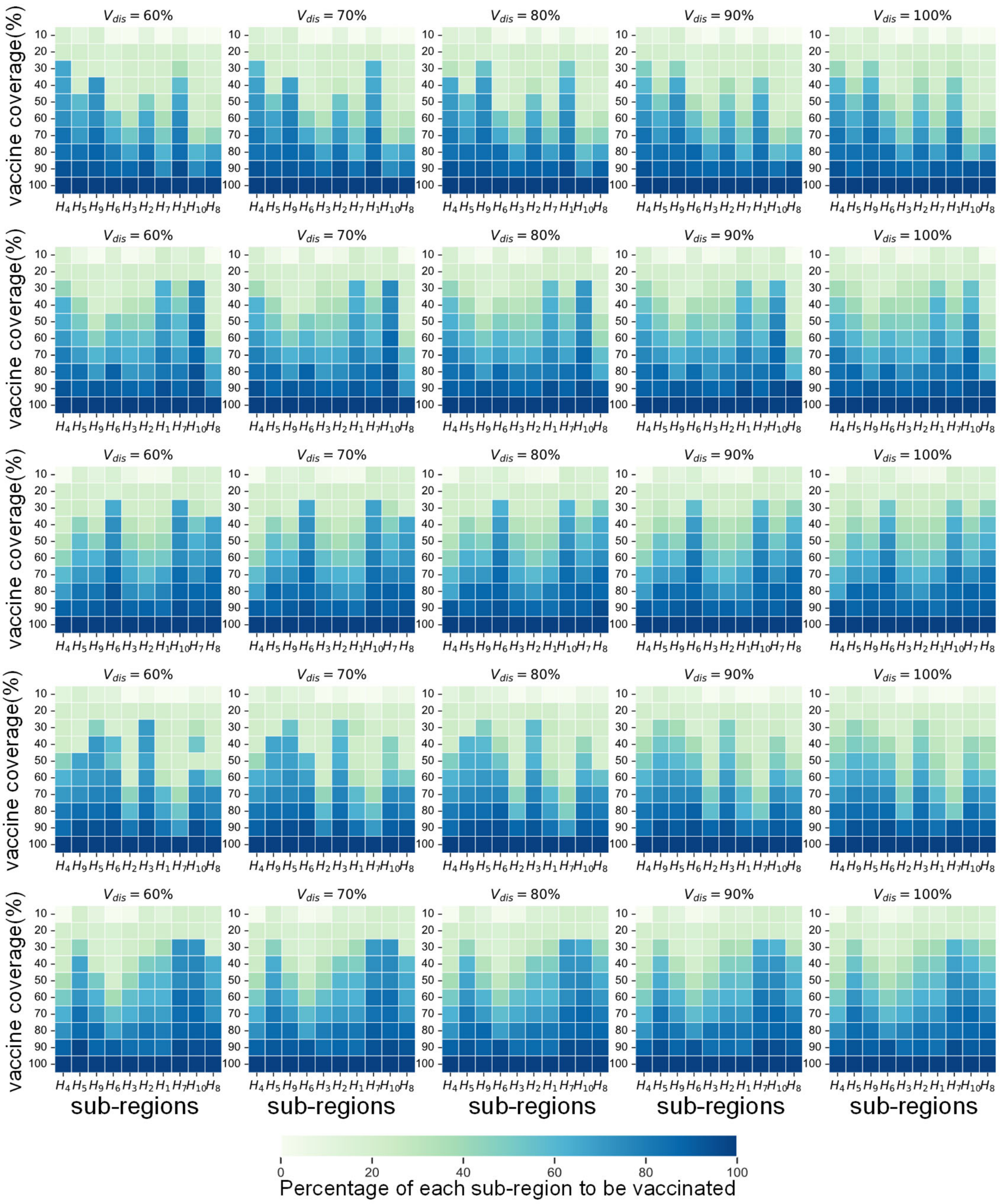

Figure S28: Near-optimal allocation strategies to minimize deaths under scenario 2 and low-intensity non-pharmacological interventions. For each heat map, each row from left to right is the decreasing direction of transmission risk, representing the total vaccine supply (percentage of the total population vaccinated) and each column represents a different subregion. Colors represent the percentage of the population in a sub-region to be vaccinated. For the whole figure, each row represents the result of one simulation.

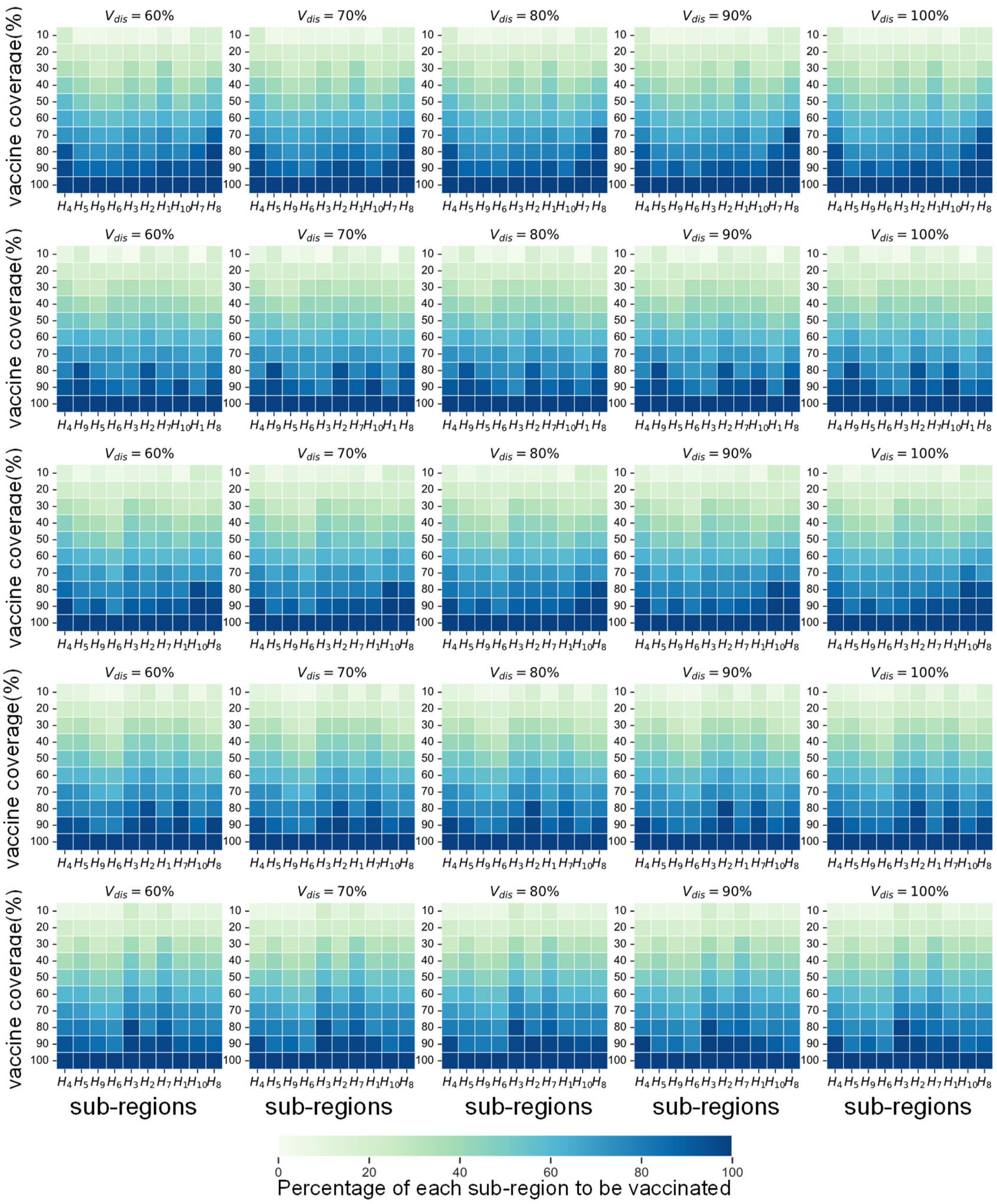

Figure S29: Near-optimal allocation strategies to minimize deaths under scenario 3 and low-intensity non-pharmacological interventions. For each heat map, each row from left to right is the decreasing direction of transmission risk, representing the total vaccine supply (percentage of the total population vaccinated) and each column represents a different subregion. Colors represent the percentage of the population in a sub-region to be vaccinated. For the whole figure, each row represents the result of one simulation.

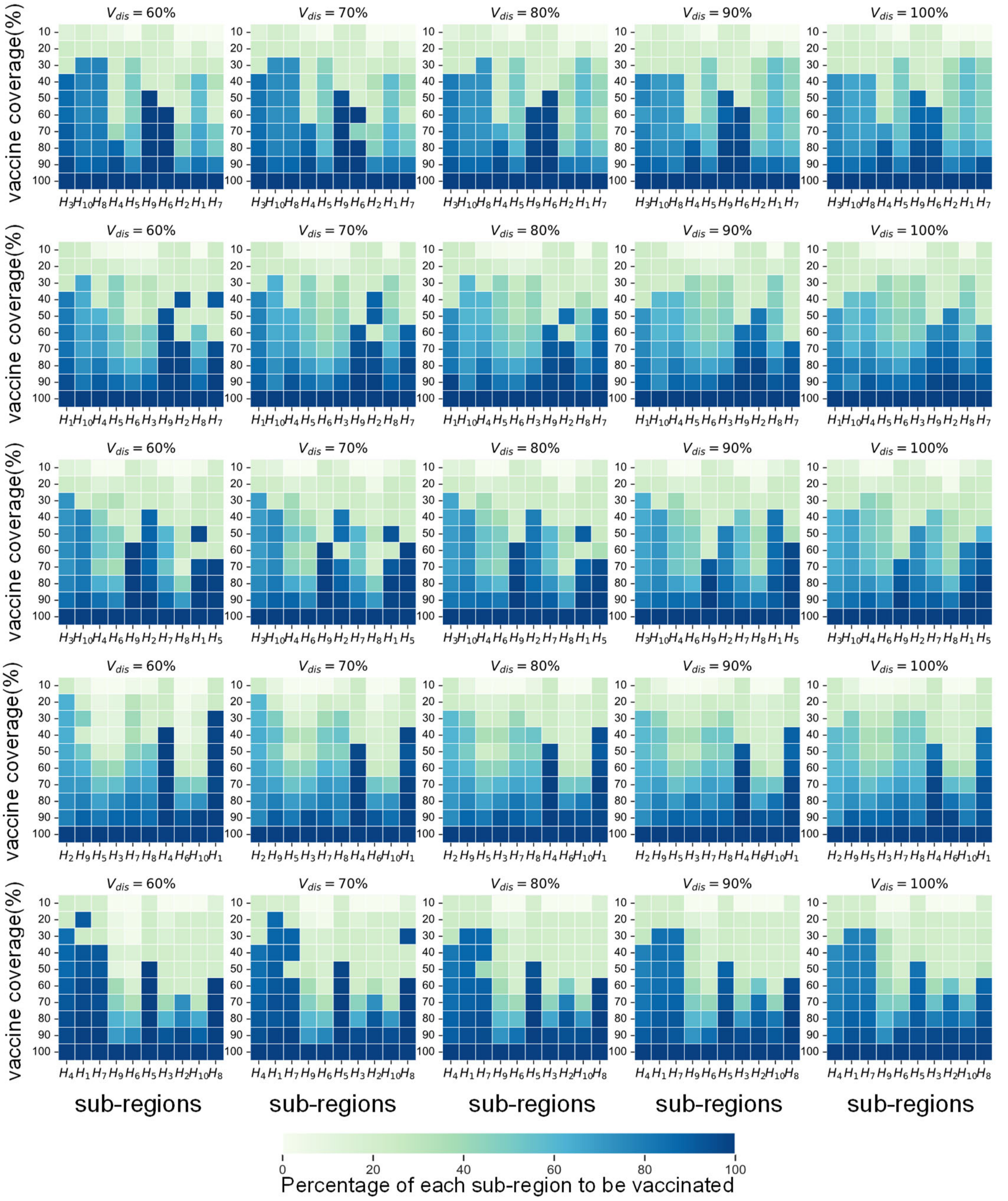

Figure S30: Near-optimal allocation strategies to minimize deaths under scenario 4 and low-intensity non-pharmacological interventions. For each heat map, each row from left to right is the decreasing direction of transmission risk, representing the total vaccine supply (percentage of the total population vaccinated) and each column represents a different subregion. Colors represent the percentage of the population in a sub-region to be vaccinated. For the whole figure, each row represents the result of one simulation.

References

[1] Johns Hopkins University and Medicine, *Coronavirus COVID-19 Global Cases by the Center for* Systems Science and Engineering (CSSE) at Johns Hopkins University (JHU)(2019). https://coronavirus.jhu.edu/map.html [Accessed July 16, 2021].

[2] Anderson S C, Edwards A M, Yerlanov M, et al. Quantifying the impact of COVID-19 control measures using a Bayesian model of physical distanc ing.PLoS computationalbiology (2020) 16:e1008274. doi:10.1371/journal.pcbi. 1008274.

[3] Cao W, Dai H, Zhu J, et al. Analysis and Evaluation of Non-Pharmaceutical Interventions on Prevention and Control of COVID-19: A Case Study of Wuhan City. ISPRS International Journal of Geo-Information (2021) 10:480. doi:10.3390/ijgi10070480.

[4] Peak C M, Kahn R, Grad Y H, et al. Individual quarantine versus active monitoring of contacts for the mitigation of COVID-19: a modelling study. The Lancet Infectious Diseases (2020) 20: 1025-1033. doi: 10.1016/s1473-3099(20)30361-3.

[5] Khamsi R. If a coronavirus vaccine arrives, can the world make enough. Nature, (2020) 580:578-580. doi:10.1038/d41586-020-01063-8.

[6] Usher A D. COVID-19 vaccines for all?. The Lancet (2020) 395:1822-182 3. doi:10.1016/s0140-6736(20)31354-4.

[7] Lazebnik T, Bunimovich-Mendrazitsky S. The Signature Features of COVID-19 Pandemic in a Hybrid Mathematical Model—Implications for Optimal Work–School Lockdown Policy. advanced theory and simulations, (2021), 4: 2000298. doi: 10.1002/adts.202000298.

[8] Bunimovich-Mendrazitsky S, Stone L. Modeling polio as a disease of development. Journal of theoretical biology, (2005), 237: 302-315. doi: 10.1016/j.jtbi.2005.04.017.

[9] Lazebnik T, Bunimovich-Mendrazitsky S, Shami L. Pandemic management by a spatio–temporal mathematical model[J]. International Journal of Nonlinear Sciences and Numerical Simulation, 2021. doi: 10.1515/ijnsns-2021-0063.

[10] Viguerie A, Lorenzo G, Auricchio F, et al. Simulating the spread of COVI D-19 via aspatially- resolved susceptible–exposed–infected–recovered–deceased (SEIRD) model with heterogeneous diffusion. Applied Mathematics Letters, (2021), 111:106617. doi: 10.1016/j.aml.2020.106617.

[11] Bubar K M, Reinholt K, Kissler S M, et al. Model-informed COVID-19 vaccine prioritizationstrategies by age and serostatus. Science, (2021) 371:916-921. d oi: 10.1101/2020.09.08.20190629.

[12] Foy B H, Wahl B, Mehta K, et al. Comparing COVID-19 vaccine allocation strategies in India: A mathematical modelling study. International Journal of Infectious Diseases (2021) 103:431-438. doi: 10.1016/j.ijid.2020.12.075.

[13] Lazebnik T, Alexi A. Comparison of pandemic intervention policies in several building types using heterogeneous population model. Communications in

Nonlinear Science and Numerical Simulation, (2022), 107: 106176. doi: 10.1016/j.cnsns.2021.106176.
[14] Dagan N, Barda N, Kepten E, et al. BNT162b2 mRNA Covid-19 vaccine in a nationwide mass vaccination setting. New England Journal of Medicine (2021) 384:1412-1423. doi: 10.1056/nejmc2104281.
[15] Tartof S Y, Slezak J M, Fischer H, et al. Effectiveness of mRNA BNT162b2 COVID-19 vaccine up to 6 months in a large integrated health system in the USA: a retrospective cohort study. The Lancet (2021) 398:1407-1416. doi: 10.1016/s0140-6736(21)02183-8.
[16] Voysey M, Clemens S A C, Madhi S A, et al. Single-dose administration and the influence of the timing of the booster dose on immunogenicity and efficacy of ChAdOx1 nCoV-19 (AZD1222) vaccine: a pooled analysis of four randomised trials. The Lancet (2021) 397:881-891. doi: 10.1016/S0140-6736(21)00432-3.
[17] Baden L R, El Sahly H M, Essink B, et al. Efficacy and safety of the mRNA-1273 SARS-CoV-2 vaccine. New England Journal of Medicine (2021) 384:403-416.
[18] Jara A, Undurraga E A, González C, et al. Effectiveness of an inactivated SARS-CoV-2 vaccine in Chile. New England Journal of Medicine (2021) 385:875-884.
[19] Jentsch P C, Anand M, Bauch C T. Prioritising COVID-19 vaccination in changing social and epidemiological landscapes: a mathematical modelling study. The Lancet Infectious Diseases (2021). doi: 10.1016/s1473-3099(21)00057-8.
[20] Matrajt L, Eaton J, Leung T, et al. Optimizing vaccine allocation for COVID-19 vaccines shows the potential role of single-dose vaccination. Nature communications (2021)12: 1-18. doi: 10.1101/2020.09.08.20190629.
[21] Matrajt L, Eaton J, Leung T, et al. Vaccine optimization for COVID-19: Who to vaccinate first?. Science Advances (2021)7:abf1374. doi:10.1101/2020.08.14.20175257.
[22] Araz O M, Galvani A, Meyers L A. Geographic prioritization of distributing pandemic influenza vaccines. Health Care Management Science (2012) 15:1 75-187. doi:10.1007/s10729-012-9199-6.
[23] Azman A S, Lessler J. Reactive vaccination in the presence of disease hotspots. Proceedings of the Royal Society B: Biological Sciences (2015)282:2014134 1. doi:10.1098/rspb.2014.1341.
[24] Wu J T, Riley S, Leung G M. Spatial considerations for the allocation of pre-pandemic influenza vaccination in the United States. Proceedings of the Royal Society B: Biological Sciences (2007) 274:2811-2817. doi: 10.1098/rspb.2007.0893.
[25] Krzysztofowicz S, Osińska-Skotak K. The use of GIS technology to optimize COVID-19 vaccine distribution: a case study of the city of Warsaw, Poland. International Journal of Environmental Research and Public Health, (2021), 18: 5636. doi: 10.3390/ijerph18115636.
[26] Zhou S, Zhou S, Zheng Z, et al. Optimizing spatial allocation of COVID-19 vaccine by agent-based spatiotemporal simulations. GeoHealth (2021)5:e202

1GH000427. doi:10.1029/2021gh000427.
[27] Venkatramanan S, Chen J, Fadikar A, et al. Optimizing spatial allocation of seasonal influenza vaccine under temporal constraints. PLoS computational biology (2019) 15:e1007111. doi:10.1371/journal.pcbi.1007111.
[28] Molla J, Ponce de León Chávez A, Hiraoka T, et al. Adaptive and optimized COVID-19 vaccination strategies across geographical regions and age groups. PLoS computational biology, 2022, 18(4): e1009974. doi: 10.1371/journal.pcbi.1009974.
[29] Joseph C. Lemaitre, Damiano Pasetto, Mario Zanon, Enrico Bertuzzo, Lorenzo Mari, Stefano Miccoli, Renato Casagrandi, Marino Gatto, Andrea Rinaldo. Optimal control of the spatial allocation of COVID-19 vaccines: Italy as a case study. medRxiv 2021.05.06.21256732. doi: 10.1101/2021.05.06.21256732.
[30] Kojima N, Klausner J D. Protective immunity after recovery from SARS-CoV-2 infection. The Lancet infectious diseases (2022) 22:12-14. doi: 10.1016/s1473-3099(21)00676-9.
[31] Sariol A, Perlman S. Lessons for COVID-19 immunity from other coronavirus infections. Immunity (2020) 53:248-263. doi: 10.1016/j.immuni.2020.07.005.
[32] Prem K, Cook A R, Jit M. Projecting social contact matrices in 152 countries using contactsurveys and demographic data. PLoS computational biology (2017) 13:e1005697. doi: 10.1371/journal.pcbi.1005697.
[33] Arregui S, Aleta A, Sanz J, et al. Projecting social contact matrices to different demographicstructures. PLoS computational biology, (2018) 14:e1006638. doi: 10.1371/journal.pcbi.1006638.
[34] CDC,Population.Projections. https://wonder.cdc.gov/wonder/help/populationprojections.html [accessed 26 June 2021].
[35] Mehrotra D V, Janes H E, Fleming T R, et al. Clinical endpoints for evaluati ng efficacy in COVID-19 vaccine trials. Annals of internal medicine (2021) 174:221-228.
[36] Van den Driessche P, Watmough J. Reproduction numbers and sub-threshol d endemic equilibria for compartmental models of disease transmission. Ma thematical biosciences (2002) 180: 29-48. doi: 10.1016/s0025-5564(02)00108 -6.
[37] Davies N G, Klepac P, Liu Y, et al. Age-dependent effects in the transmis sion and control of COVID-19 epidemics. Nature medicine, (2020),26:1 205-1211. doi:10.1101/2020.03.24.20043018.
[38] Laxminarayan R, Wahl B, Dudala S R, et al. Epidemiology and transmission dynamics of COVID-19 in two Indian states. Science, 2020, 370: 691-697. doi: 10.1126/science.abd7672.
[39] Long Q X, Tang X J, Shi Q L, et al. Clinical and immunological assessment of asymptomaticSARS-CoV-2 infections. Nature medicine (2020) 26:1200-1204. doi: 10.1038/s41591-020-0965-6.

[40] Khoury D S, Cromer D, Reynaldi A, et al. Neutralizing antibody levels are highly predictive of immune protection from symptomatic SARS-CoV-2 infection. Nature medicine(2021) 27:1205-1211. doi: 10.1038/s41591-021-01377-8.
